# Supplementary material for: Mortality and antibiotic timing in deep learning-derived surviving sepsis campaign risk groups: a multicenter study
Source: Crit Care. 2025 Jul 14;29:302. doi: 10.1186/s13054-025-05493-6 (PMC12257722; doi:10.1186/s13054-025-05493-6)

**SUPPLEMENT:**

Data Missingness

We used preprocessing codes to compute the percent missingness for all features (7 vital signs, and 43 laboratory results). Missing data were imputed using either time-limited carry forward or mean imputation. We excluded patients who did not have at least one vital sign documented in the previous 6 hours prior to algorithm. We also excluded patients who did not have at least one laboratory value recorded in the previous 24 hours prior to algorithm prediction.

For each of these 50 clinical features we track 1. the most recent value, 2. the TSLM (time since last measurement of this feature), 3. the baseline value of this feature, and 4. the delta value (rate of change between the last two measurements) of the feature. Since each Shock-Net considers each time point it analyzes separately, these three additional values for each feature allow our model to take temporal trends into consideration when making predictions.

We report the missingness for each clinical feature, by study site, in Supplemental Tables 1 and 2.

**Supplemental Table 1:** Vital Sign Missingness (%)

| Vital | Development Site | Validation Site |
| --- | --- | --- |
| Heart rate | 0.1 | 0.1 |
| Oxygen saturation (spO2) | 0.6 | 0.5 |
| Temperature | 16.3 | 16.2 |
| Systolic blood pressure | 0.1 | 0.2 |
| Mean arterial blood pressure | 20.1 | 15.4 |
| Diastolic blood pressure | 0.1 | 0.2 |
| Respiratory rate | 1.5 | 2.1 |

**Supplemental Table 2:** Lab Missingness (%)

| Lab Missingness | Development Site | Validation Site |
| --- | --- | --- |
| End-tidal carbon dioxide | 92.5 | 93.8 |
| Base excess (on blood gas) | 96.7 | 67.4 |
| Bicarbonate (on blood gas) | 10.5 | 100 |
| FiO2 (on blood gas) | 97.5 | 78.4 |
| PH (on blood gas) | 96.7 | 96.2 |
| PaCO2 | 96.8 | 96.4 |
| SaO2 | 96.9 | 67.8 |
| AST | 18 | 17.9 |
| BUN | 10.6 | 6.9 |
| Alkaline phosphatase | 17.9 | 16.1 |
| Calcium | 10.5 | 3.8 |
| Chloride | 10.5 | 6.9 |
| Creatinine | 10.6 | 7.1 |
| Bilirubin Direct | 96.1 | 96.4 |
| Glucose | 10.7 | 7.1 |
| Lactate | 37.8 | 37.2 |
| Magnesium | 65.8 | 57 |
| Phosphate | 77.3 | 67.4 |
| Potassium | 10.5 | 8.7 |
| Bilirubin Total | 19.8 | 16.1 |
| TroponinI | 80.4 | 100 |
| Hematocrit | 11.7 | 10.5 |
| Hemoglobin | 11 | 10.5 |
| PTT | 72.8 | 46.9 |
| White blood cell count | 11.7 | 10.6 |
| Fibrinogen | 99.4 | 99.3 |
| Platelets | 12 | 11 |
| PaO2 | 96.7 | 96.2 |
| BNP | 82.7 | 100 |
| Sodium | 11.3 | 11.7 |
| Procalcitonin | 89 | 98.8 |
| CRP | 100 | 94 |
| Ammonia | 95.6 | 91.2 |
| Hemoglobin A1C | 99.6 | 99.5 |
| lymphocytes | 14.3 | 100 |
| Lymphocytes (differential) | 12 | 33.3 |
| Albumin | 18.5 | 22 |
| ALT | 19.1 | 18.2 |
| D dimer | 96.4 | 99.7 |
| Ferritin | 99.9 | 99 |
| Red cell distribution width | 11.8 | 10.5 |
| Sedimentation rate | 94.1 | 95.1 |
| LDH | 98.2 | 98.8 |

**Supplemental Table 3: Other Variables Used in COMPOSER and SHOCK-NET**

Here we list the non-clinical variables used in our DL models (in addition to those in Supplemental Tables 1 and 2). All of the variables used in our model are described in detail in the supplement of the previously published DETERIO model^1.^

| Non-Clinical Features | | |
| --- | --- | --- |
| Age | RESPSOFA | on_anticonvulsants |
| Gender | coaSOFA | on_antipsychotics |
| MICU admission | liverSOFA | on_bleed_reversal |
| SICU admission | CVSSOFA | on_vasodilator |
| preLOS | perfusionSOFA | on_antihypertensive |
| ICULOS | renalSOFA | on_antiarrhythmic |
| TempSIRS | CMSsofa | on_prostacyclin |
| RESPSIRS | on_vent | on_neuro_block |
| HRSIRS | on_anesthesia | on_pain_med |
| WBCSIRS | on_anticoagulants | on_steroids |

**Supplemental Table 4:** ICD Code Variables in COMPOSER and Shock-Net

This table contains patient features described by the ICD codes in our data. These variables are described in detail in the supplement of the previously published DETERIO model^1.^

| ICD Features | | |
| --- | --- | --- |
| has_intravascular_coagulation | has_cerebral_infarction | has_hiv |
| has_necrotizing_fasciitis | has_meningitis | has_hypertension |
| has_tumor_lysis_syndrome | has_malignant_liver_cancer | has_obesity |
| has_shock | has_gi_hemorrhage | has_brain_condition |
| has_metastatic_cancer | has_acute_renal_failure | has_pulmonary_condition |
| has_stemi | has_chronic_liver_disease | has_valve_disease |
| has_myeloblastic_leukemia | has_neutropenia | has_uti |
| has_liver_failure | has_cardiac_arrythmia | has_anemia |
| has_malignant_pleural_effusion | has_malnutrition | has_pneumonia |
| has_pneumothorax | has_pulmonary_heart_disease | has_hyperlipidemia |
| has_cachexia | has_ckd | has_depression |
| has_coma | has_plasma_protein_disorder | has_bacteremia |
| has_peritonitis | has_aplasia | has_reflux_disease |
| has_lymphoma | has_aspiration_pneumonitis | has_hypothyroidism |
| has_carditis | has_ami | has_insulin |
| has_septic_empolism | has_chf | Liver cirrhosis |
| has_coagulation_defect | has_encephalopathy | Immune conditions |
| has_primary_lung_cancer | has_thrombocytopenia | Solid malignancy |
| has_lymphoid_leukemia | has_coronary_artery_disease | Organ transplant |
| has_pulmonary_embolism | has_copd | Rheumatologic/  Inflammatory |
| has_pericardial_effusion | has_diabetes |  |

DL Model Performance

To evaluate the performance of Shock-Net and COMPOSER we display the confusion matrix for patient stratification at both the development site and the validation site. These show how our predictive models differed from the clinical labels we use as the ground truth to train and test our models against.

We consider the clinical ground truth labels the ’true’ labels and the output of our DL models as ‘predicted’ labels, and we tabulate how many patients there are with each label combination. We perform this analysis for the development site (Supplementary Figure 1) and validation site (Supplementary Figure 2) for all patients and excluding patients who develop shock within 3 hours of ED triage (Supplementary Figures 3 & 4 ).

**Supplemental Figure 1.** Confusion matrix for the development site patients analyzed by our DL models. Each cell in the matrix displays the total number of patients with a given combination of true and predicted labels (top) and the corresponding percentage of patients within each true label category (bottom).

**Supplemental Figure 2.** Confusion matrix for the validation site patients analyzed by our DL models. Each cell in the matrix displays the total number of patients with a given combination of true and predicted labels (top) and the corresponding percentage of patients within each true label category (bottom).

**Supplemental Figure 3:** Confusion Matrix for the development site patients analyzed by our DL models, excluding patients who develop shock within 3 hours of ED triage. Each cell in the matrix displays the total number of patients with a given combination of true and predicted labels (top) and the corresponding percentage of patients within each true label category (bottom).

**Supplemental Figure 4:** Confusion Matrix for the validation site patients analyzed by our DL models, excluding patients who develop shock within 3 hours of ED triage. Each cell in the matrix displays the total number of patients with a given combination of true and predicted labels (top) and the corresponding percentage of patients within each true label category (bottom).

Shock-Net Model Validation

For our external validation on UCI patient data (consisting of 4776 patients), we find that our PPV is 20%, and our NPV is 97.9% when the sensitivity per patient is set to 83%. In this test our specificity per patient is 74%, with a prevalence (of shock) of 7.5%.

We run the same sample size diagnostic algorithm that was used with COMPOSER validation data to ensure that the Shock-Net validation data sample size was sufficient to claim such PPV and NPV^2^. This test was run with a confidence of 95% (alpha = 0.05) and a power of 80% (beta = 0.2), as was done in the previous COMPOSER analysis. We find that the sample size needed to make these claims is approximately 19,000 patients. Our combined dataset meets this requirement.

Detailed Antibiotic Timing Data For All Patients

**Supplemental Table 5:** Development Antibiotic Timing Among Patients Treated with Suspected Sepsis Infection Between Jan 2016 and Dec 2023.

| **Variables** | **Group 1**  **Shock Likely to Develop and Sepsis Probable**  **(n=3645)** | **Group 2**  **Shock Likely to Develop and Sepsis Possible**  **(n=124)** | **Group 3**  **Shock Unlikely to Develop and Sepsis Probable**  **(n=14761)** | **Group 4**  **Shock Unlikely to Develop and Sepsis Possible**  **(n=11235)** | **P-Value** |
| --- | --- | --- | --- | --- | --- |
| **Antibiotic Timing** | | | | |  |
| Received Abx, Anytime % (n) | 95.5 (3482/3645) | 89.5 (111/124) | 87.4 (12904/14761) | 75.7 (8507/11235) | < 0.001 |
| Time to Abx from triage [IQR] ^a^ | 1.7 [1.0 - 3.1] | 3.0 [1.7 - 6.2] | 2.8 [1.5 - 5.1] | 4.6 [2.7 - 8.0] | < 0.001 |
| Time to Abx from T0 [IQR] *^a,b^* | 1.0 [0.5 - 2.0] | 1.8 [0.7 - 4.2] | 1.1 [0.6 - 2.5] | 1.0 [0.5 - 2.8] | < 0.001 |
| Received Abx <1 hr triage, % (n) | 25.5 (931/3645) | 8.1  (10/124) | 12.3 (1820/14761) | 3.5  (396/11235) | < 0.001 |
| Received Abx 1-3 hrs triage, % (n) | 45.1 (1644/3645) | 35.5  (44/124) | 35.0 (5164/14761) | 18.6 (2091/11235) | < 0.001 |
| Received Abx >3 hrs triage, % (n) | 24.9 (907/3645) | 46.0  (57/124) | 40.1 (5920/14761) | 53.6 (6020/11235) | < 0.001 |
| Received Abx <1 hr T0, % (n) *^b^* | 47.2 (1722/3645) | 37.1  (46/124) | 39.3 (5801/14761) | 36.8 (4138/11235) | < 0.001 |
| Received Abx 1-3 hrs T0, % (n) *^b^* | 34.5 (1258/3645) | 23.4  (29/124) | 30.5 (4507/14761) | 20.7 (2326/11235) | < 0.001 |
| Received Abx >3 hrs T0, % (n) *^b^* | 13.8 (502/3645) | 29.0 (36/124) | 17.6 (2596/14761) | 18.2 (2043/11235) | < 0.001 |
| **Short term Mortality*^c^*** | | | | |  |
| Didn’t Receive Abx, % (n) | 22.7 (37/163) | 7.7 (1/13) | 1.5 (28/1857) | 0.6 (16/2728) | < 0.001 |

*^a^* Median time in hours. *^b^* T0 = time of clinical suspicion of potential sepsis. *^c^* Composite of in-hospital mortality and discharge to hospice. Hr(s), hours. SOFA, sequential organ failure assessment. Abx, antibiotics

**Supplemental Table 6:** Validation Site ED Stratification, Mortality, and Antibiotic Timing Among Patients Treated with Suspected Sepsis Infection Between Jan 2023 and Oct 2024.

| **Variables** | **Group 1**  **Shock Likely to Develop and Sepsis Probable**  **(n=706)** | **Group 2**  **Shock Likely to Develop and Sepsis Possible**  **(n=51)** | **Group 3**  **Shock Unlikely to Develop and Sepsis Probable**  **(n=1633)** | **Group 4**  **Shock Unlikely to Develop and Sepsis Possible**  **(n=1932)** | **P-Value** |
| --- | --- | --- | --- | --- | --- |
| **Antibiotic Timing** | | | | | |
| Received Abx, Anytime % (n) | 94.9 (670/706) | 92.2 (47/51) | 93.9 (1533/1633) | 88.6 (1711/1932) | < 0.001 |
| Time to Abx from triage [IQR] *^a^* | 1.8 [1.0 - 3.3] | 3.1 [1.4 - 6.4] | 3.7 [1.9 - 6.9] | 6.0 [3.2 - 9.4] | < 0.001 |
| Time to Abx from T0 [IQR] *^a,b^* | 1.1 [0.6 - 1.9] | 1.0 [0.5 - 2.6] | 1.0 [0.6 - 2.1] | 0.9 [0.5 - 1.8] | < 0.001 |
| Received Abx <1 hr triage, % (n) | 24.6 (174/706) | 9.8 (5/51) | 11.0 (179/1633) | 6.3  (122/1932) | < 0.001 |
| Received Abx 1-3 hrs triage, % (n) | 42.4 (299/706) | 35.3 (18/51) | 28.2 (461/1633) | 14.4  (279/1932) | < 0.001 |
| Received Abx >3 hrs triage, % (n) | 27.9 (197/706) | 47.1 (24/51) | 54.7 (893/1633) | 67.8 (1310/1932) | < 0.001 |
| Received Abx <1 hr T0, % (n) *^b^* | 43.9 (310/706) | 47.1  (24/51) | 44.5 (726/1633) | 47.7  (921/1932) | 0.175 |
| Received Abx 1-3 hrs T0, % (n) *^b^* | 37.7 (266/706) | 25.5 (13/51) | 35.2 (574/1633) | 28.2  (544/1932) | < 0.001 |
| Received Abx >3 hrs T0, % (n) *^b^* | 13.3  (94/706) | 19.6  (10/51) | 14.3 (233/1633) | 12.7  (246/1932) | 0.327 |
| **Short term Mortality*^c^*** | | | | | |
| Didn’t Receive Abx, % (n) | 27.8 (10/36) | 0.0 (0/4) | 3.0 (3/100) | 1.4 (3/221) | < 0.001 |

*^a^* Median time in hours. *^b^* T0 = time of clinical suspicion of potential sepsis. *^c^* Composite of in-hospital mortality and discharge to hospice. Hr(s), hours. SOFA, sequential organ failure assessment. Abx, antibiotics

Sub-analysis Excluding Patients Who Developed Shock Within 3 Hours of ED Triage

**Supplemental Table 7:** Development Site ED Stratification, Mortality, and Antibiotic Timing Among Patients Treated with Suspected Sepsis Infection Between Jan 2016 and Dec 2023. Patients who develop shock within 3 hours of triage are excluded from this analysis.

| **Variables** | **Group 1 Shock Likely to Develop and Sepsis Probable**  **(n=2554)** | **Group 2**  **Shock Likely to Develop and Sepsis Possible**  **(n=94)** | **Group 3**  **Shock Unlikely to Develop and Sepsis Probable**  **(n=14417)** | **Group 4**  **Shock Unlikely to Develop and Sepsis Possible**  **(n=11168)** | **P-Value** |
| --- | --- | --- | --- | --- | --- |
| **Patient Characteristics** | | | | | |
| Age Yrs [IQR] | 64 [53 - 75] | 61 [52 - 71] | 61 [48 - 72] | 58 [42 - 69] | < 0.001 |
| Female % (n) | 40.4 (1032/2554) | 40.4 (38/94) | 39.8 (5739/14417) | 47.6 (5316/11168) | < 0.001 |
| Black Race % (n) | 9.6 (244/2554) | 14.9 (14/94) | 11.3 (1626/14417) | 13.5 (1511/11168) | < 0.001 |
| White Race % (n) | 51.4 (1312/2554) | 51.1 (48/94) | 51.3 (7397/14417) | 50.8 (5678/11168) | 0.894 |
| Asian Race % (n) | 8.6 (219/2554) | 8.5 (8/94) | 7.9 (1138/14417) | 6.6 (741/11168) | < 0.001 |
| Charlson Comorbidity Index [IQR] | 2 [1 - 5] | 1 [0 - 3] | 2 [1 - 5] | 2 [0 - 4] | < 0.001 |
| SOFA [IQR] | 2 [1 - 4] | 2 [1 - 4] | 1 [0 - 2] | 0 [0 - 1] | < 0.001 |
| Confirmed Sepsis*^a^*, % (n) | 62.3 (1592/2554) | 46.8 (44/94) | 34.5 (4970/14417) | 14.2 (1587/11168) | < 0.001 |
| Developed Shock, % (n) | 20.3 (518/2554) | 16.0 (15/94) | 3.8 (544/14417) | 1.0 (115/11168) | < 0.001 |
| **Antibiotic Timing** | | | | | |
| Received Abx, Anytime % (n) | 93.6 (2391/2554) | 86.2 (81/94) | 87.1 (12560/14417) | 75.6 (8440/11168) | < 0.001 |
| Time to Abx from triage [IQR] *^b^* | 1.8 [1.0 - 3.4] | 3.2 [1.8 - 7.0] | 2.8 [1.5 - 5.1] | 4.6 [2.7 - 8.0] | < 0.001 |
| Time to Abx from T0 [IQR] *^b,c^* | 1.1 [0.6 - 2.1] | 1.7 [0.6 - 4.8] | 1.2 [0.6 - 2.5] | 1.0 [0.5 - 2.8] | < 0.001 |
| Received Abx <1 hr triage, % (n) | 21.8 (557/2554) | 6.4 (6/94) | 12.0 (1730/14417) | 3.5 (393/11168) | < 0.001 |
| Received Abx 1-3 hrs triage, % (n) | 44.2 (1128/2554) | 31.9 (30/94) | 34.7 (5003/14417) | 18.5 (2064/11168) | < 0.001 |
| Received Abx >3 hrs triage, % (n) | 27.6 (706/2554) | 47.9 (45/94) | 40.4 (5827/14417) | 53.6 (5983/11168) | < 0.001 |
| Received Abx <1 hr T0, % (n) *^b^* | 44.2 (1130/2554) | 35.1 (33/94) | 39.0 (5620/14417) | 37.0 (4130/11168) | < 0.001 |
| Received Abx 1-3 hrs T0, % (n) *^b^* | 34.7 (887/2554) | 20.2 (19/94) | 30.5 (4390/14417) | 20.5 (2293/11168) | < 0.001 |
| Received Abx >3 hrs T0, % (n) *^b^* | 14.6 (374/2554) | 30.9 (29/94) | 17.7 (2550/14417) | 18.1 (2017/11168) | < 0.001 |
| **Short term Mortality*^d^*** | | | | | |
| Overall, % (n) | 18.1 (463/2554) | 13.8 (13/94) | 4.7 (673/14417) | 1.8 (205/11168) | < 0.001 |
| Received Abx <1 hr triage, % (n) | 14.2 (79/557) | 16.7 (1/6) | 3.7 (64/1730) | 1.0 (4/393) | < 0.001 |
| Received Abx 1-3 hrs triage, % (n) | 19.1 (215/1128) | 16.7 (5/30) | 5.1 (253/5003) | 2.3 (48/2064) | < 0.001 |
| Received Abx >3 hrs triage, % (n) | 18.7 (132/706) | 13.3 (6/45) | 5.6 (328/5827) | 2.3 (137/5983) | < 0.001 |
| Received Abx <1 hr T0, % (n) | 17.4 (197/1130) | 18.2 (6/33) | 4.4 (249/5620) | 1.2 (50/4130) | < 0.001 |
| Received Abx 1-3 hrs T0, % (n) | 18.7 (166/887) | 10.5 (2/19) | 5.4 (239/4390) | 2.7 (61/2293) | < 0.001 |
| Received Abx >3 hrs T0, % (n) | 16.8 (63/374) | 13.8 (4/29) | 6.2 (157/2550) | 3.9 (78/2017) | < 0.001 |
| Didn’t Receive Abx, % (n) | 22.7 (37/163) | 7.7 (1/13) | 1.5 (28/1857) | 0.6 (16/2728) | < 0.001 |

*^a^* Patients who met Sepsis 3 criteria during hospitalization. *^b^* Median time in hours. *^c^* T0 = time of clinical suspicion of potential sepsis. *^d^* Composite of in-hospital mortality and discharge to hospice. Hr(s), hours. SOFA, sequential organ failure assessment. Abx, antibiotics

**Supplemental Table 8:** Repeat of Validation Site ED Stratification, Mortality, and Antibiotic Timing Among Patients Treated with Suspected Sepsis Infection Between Jan 2023 and Oct 2024. Patients who develop shock within 3 hours of triage are excluded from this analysis.

| **Variables** | **Group 1**  **Shock Likely to Develop and Sepsis Probable**  **(n=519)** | **Group 2**  **Shock Likely to Develop and Sepsis Possible**  **(n=45)** | **Group 3**  **Shock Unlikely to Develop and Sepsis Probable**  **(n=1604)** | **Group 4**  **Shock Unlikely to Develop and Sepsis Possible**  **(n=1924)** | **P-Value** |
| --- | --- | --- | --- | --- | --- |
| **Patient Characteristics** | | | | |  |
| Age Yrs [IQR] | 67 [55 - 80] | 68 [49 - 78] | 59 [43 - 73] | 56 [37 - 71] | < 0.001 |
| Female % (n) | 43.7 (227/519) | 28.9 (13/45) | 44.1 (708/1604) | 47.8 (920/1924) | 0.012 |
| Black Race % (n) | 1.3 (7/519) | 2.2 (1/45) | 2.4 (38/1604) | 3.5 (68/1924) | 0.029 |
| White Race % (n) | 42.6 (221/519) | 44.4 (20/45) | 45.0 (721/1604) | 45.8 (882/1924) | 0.62 |
| Asian Race % (n) | 24.9 (129/519) | 31.1 (14/45) | 20.3 (326/1604) | 17.7 (340/1924) | < 0.001 |
| Charlson Comorbidity Index [IQR] | 1 [0 - 3] | 1 [0 - 2] | 1 [0 - 3] | 1 [0 - 2] | < 0.001 |
| SOFA [IQR] | 2 [1 - 4] | 2 [1 - 4] | 1 [0 - 2] | 0 [0 - 1] | < 0.001 |
| Confirmed Sepsis*^a^*, % (n) | 61.5 (319/519) | 35.6 (16/45) | 33.8 (542/1604) | 12.6 (242/1924) | < 0.001 |
| Developed Shock, % (n) | 14.8 (77/519) | 8.9 (4/45) | 3.5 (56/1604) | 0.8 (16/1924) | < 0.001 |
| **Antibiotic Timing** | | | | |  |
| Received Abx, Anytime % (n) | 93.1 (483/519) | 91.1 (41/45) | 93.8 (1504/1604) | 88.5 (1703/1924) | < 0.001 |
| Time to Abx from triage [IQR] *^b^* | 2.1 [1.0 - 3.6] | 3.7 [1.6 - 7.1] | 3.7 [1.9 - 7.0] | 6.0 [3.2 - 9.4] | < 0.001 |
| Time to Abx from T0 [IQR] *^b,c^* | 1.1 [0.6 - 2.0] | 0.9 [0.5 - 2.7] | 1.0 [0.6 - 2.2] | 0.9 [0.5 - 1.8] | < 0.001 |
| Received Abx <1 hr triage, % (n) | 21.6 (112/519) | 8.9 (4/45) | 10.7 (172/1604) | 6.3 (121/1924) | < 0.001 |
| Received Abx 1-3 hrs triage, % (n) | 40.1 (208/519) | 31.1 (14/45) | 28.1 (450/1604) | 14.4 (277/1924) | < 0.001 |
| Received Abx >3 hrs triage, % (n) | 31.4 (163/519) | 51.1 (23/45) | 55.0 (882/1604) | 67.8 (1305/1924) | < 0.001 |
| Received Abx <1 hr T0, % (n) *^b^* | 41.6 (216/519) | 48.9 (22/45) | 44.5 (713/1604) | 47.5 (914/1924) | 0.066 |
| Received Abx 1-3 hrs T0, % (n) *^b^* | 36.8 (191/519) | 22.2 (10/45) | 34.9 (560/1604) | 28.2 (543/1924) | < 0.001 |
| Received Abx >3 hrs T0, % (n) *^b^* | 14.6 (76/519) | 20.0 (9/45) | 14.4 (231/1604) | 12.8 (246/1924) | 0.271 |
| **Short term Mortality*^d^*** | | | | |  |
| Overall, % (n) | 24.3 (126/519) | 8.9 (4/45) | 8.0 (128/1604) | 2.8 (54/1924) | < 0.001 |
| Received Abx <1 hr triage, % (n) | 22.3 (25/112) | 0.0 (0/4) | 6.4 (11/172) | 4.1 (5/121) | < 0.001 |
| Received Abx 1-3 hrs triage, % (n) | 26.4 (55/208) | 7.1 (1/14) | 7.6 (34/450) | 4.3 (12/277) | < 0.001 |
| Received Abx >3 hrs triage, % (n) | 22.1 (36/163) | 13.0 (3/23) | 9.1 (80/882) | 2.6 (34/1305) | < 0.001 |
| Received Abx <1 hr T0, % (n) | 23.1 (50/216) | 13.6 (3/22) | 6.7 (48/713) | 2.1 (19/914) | < 0.001 |
| Received Abx 1-3 hrs T0, % (n) | 26.7 (51/191) | 0.0 (0/10) | 7.1 (40/560) | 4.1 (22/543) | < 0.001 |
| Received Abx >3 hrs T0, % (n) | 19.7 (15/76) | 11.1 (1/9) | 16.0 (37/231) | 4.1 (10/246) | < 0.001 |
| Didn’t Receive Abx, % (n) | 27.8 (10/36) | 0.0 (0/4) | 3.0 (3/100) | 1.4 (3/221) | < 0.001 |

*^a^* Patients who met Sepsis 3 criteria during hospitalization. *^b^* Median time in hours. *^c^* T0 = time of clinical suspicion of potential sepsis. *^d^* Composite of in-hospital mortality and discharge to hospice. Hr(s), hours. SOFA, sequential organ failure assessment. Abx, antibiotics

Short Term Mortality by Risk Group and Antibiotic Timing, Unadjusted for Baseline Characteristics:

By sub-stratifying each risk group into those who received antibiotics within 1 hour of triage, within 1-3 hours of triage, and those who received antibiotics more than 3 hours after triage, we assessed whether early antibiotics significantly decreased mortality within each risk group and whether delayed antibiotics significantly increased mortality within each risk group. These hypotheses were tested using one-sided Pearson’s Chi-square test for independence. First, we applied this Chi-squared test to see if the mortality of those who received antibiotics within an hour of triage in each risk group were significantly lower than the other patients within the risk group. Then we applied this Chi-squared test to see if those who received antibiotics after 3 hours of triage had significantly higher mortality compared to the other patients within the risk group. A p-value < 0.05 was considered significant for these one-sided Chi-squared tests.

Even without adjusting for age, sex, SOFA, and CCI as in Tables 3 & 4, we note that the differences in short-term mortality among those in Group 4 (Shock Unlikely to Develop and Sepsis Possible) is not significant (Supplemental Table 9). This result remains the unchanged when we exclude patients who develop shock within 3 hours of triage (Supplemental Table 10)

| **Supplemental Table 9:** Short Term Mortality by Sepsis Risk Group, Antibiotic Timing and Study Site, Unadjusted for Baseline Characteristics | | | | | |
| --- | --- | --- | --- | --- | --- |
|  | **Antibiotic Timing (From Triage)** | | |  |  |
| **Short Term Mortality, % (n)** | **<1 hour** | **1-3 hours** | **>3 hours** | **p-value 1^a^** | **p-value 2^b^** |
| **Group 1 [Shock Likely to Develop and Sepsis Probable]** | | | | | |
| **Development Site** | 20.4 (190/931) | 24.7 (406/1644) | 23.4 (212/907) | 0.010 | 0.462 |
| **Validation Site** | 26.4  (46/174) | 30.7  (92/299) | 27.4  (54/197) | 0.274 | 0.643 |
| **Group 2 [Shock Likely to Develop and Sepsis Possible]** | | | | | |
| **Development Site** | 20.0  (2/10) | 20.5  (9/44) | 17.5  (10/57) | 0.500 | 0.555 |
| **Validation Site** | 20.0  (1/5) | 22.2  (4/18) | 12.5  (3/24) | 0.500 | 0.675 |
| **Group 3 [Shock Unlikely to Develop and Sepsis Probable]** | | | | | |
| **Development Site** | 4.2 (77/1820) | 5.6 (288/5164) | 5.9 (349/5920) | 0.005 | 0.053 |
| **Validation Site** | 6.7  (12/179) | 8.7  (40/461) | 9.2  (82/893) | 0.194 | 0.264 |
| **Group 4 [Shock Unlikely to Develop and Sepsis Possible]** | | | | | |
| **Development Site** | 1.0  (4/396) | 2.4 (51/2091) | 2.4 (144/6020) | 0.052 | 0.336 |
| **Validation Site** | 4.1  (5/122) | 5.0  (14/279) | 2.7  (35/1310) | 0.636 | 0.972 |
| ^a^One sided Chi-squared test to determine if the mortality of those who received antibiotics within an hour of triage in each risk group were significantly lower than the other patients within the risk group ^b^One-sided Chi-squared test to determine if those who received antibiotics after 3 hours of triage had significantly higher mortality compared to the other patients within the risk group | | | | | |

| **Supplemental Table 10:** Short Term Mortality by Sepsis Risk Group, Antibiotic Timing and Study Site, Excluding Patients Who Develop Shock within 3 Hours of Triage, Unadjusted for Baseline Characteristics | | | | | |
| --- | --- | --- | --- | --- | --- |
|  | **Antibiotic Timing (From Triage)** | | |  |  |
| **Short Term Mortality, % (n)** | **<1 hour** | **1-3 hours** | **>3 hours** | **p-value 1^a^** | **p-value 2^b^** |
| **Group 1 [Shock Likely to Develop and Sepsis Probable)** | | | | | |
| **Development Site** | 14.2  (79/557) | 19.1  (215/1128) | 18.7  (132/706) | 0.006 | 0.252 |
| **Validation Site** | 22.3  (25/112) | 26.4  (55/208) | 22.1  (36/163) | 0.362 | 0.725 |
| **Group 2 [Shock Likely to Develop and Sepsis Possible]** | | | | | |
| **Development Site** | 16.7  (1/6) | 16.7  (5/30) | 13.3  (6/45) | 0.500 | 0.542 |
| **Validation Site** | 0.0  (0/4) | 7.1  (1/14) | 13.0  (3/23) | 0.500 | 0.393 |
| **Group 3 [Shock Unlikely to Develop and Sepsis Probable]** | | | | | |
| **Development Site** | 3.7  (64/1730) | 5.1  (253/5003) | 5.6  (328/5827) | 0.002 | 0.011 |
| **Validation Site** | 6.4  (11/172) | 7.6  (34/450) | 9.1  (80/882) | 0.206 | 0.120 |
| **Group 4 [Shock Unlikely to Develop and Sepsis Possible]** | | | | | |
| **Development Site** | 1.0  (4/393) | 2.3  (48/2064) | 2.3  (137/5983) | 0.067 | 0.342 |
| **Validation Site** | 4.1  (5/121) | 4.3  (12/277) | 2.6  (34/1305) | 0.686 | 0.938 |
| ^a^One sided Chi-squared test to determine if the mortality of those who received antibiotics within an hour of triage in each risk group were significantly lower than the other patients within the risk group ^b^One-sided Chi-squared test to determine if those who received antibiotics after 3 hours of triage had significantly higher mortality compared to the other patients within the risk group | | | | | |

Outcomes and Baseline Characteristcs of Risk Groups by Antibiotic Timing From Triage:

**Supplemental Table 11:** Baseline Characteristics and Outcomes by Antibiotic Timing from Triage Among Patients Treated with Suspected Sepsis Infection Between Jan 2016 and Dec 2023 at the Development Site in Risk Group 1.

|  | **Antibiotic Timing (From Triage)** | | |  |
| --- | --- | --- | --- | --- |
| **Variables** | **<1 hr** | **1–3 hrs** | **>3 hrs** | **P-Value** |
| Short Term Mortality, % (n) | 14.2 (79/557) | 19.1 (215/1128) | 18.7 (132/706) | 0.037 |
| Age, [IQR] | 67 [55 - 76] | 64 [54 - 75] | 63 [52 - 75] | 0.01 |
| Female, % (n) | 37.0 (206/557) | 39.6 (447/1128) | 43.6 (308/706) | 0.05 |
| Black Race, % (n) | 8.3 (46/557) | 9.4 (106/1128) | 10.2 (72/706) | 0.501 |
| White Race, % (n) | 53.3 (297/557) | 53.2 (600/1128) | 49.0 (346/706) | 0.168 |
| Asian Race, % (n) | 9.9 (55/557) | 8.2 (92/1128) | 7.9 (56/706) | 0.403 |
| Charlson Comorbidity Index, [IQR] | 2 [1 - 5] | 2 [1 - 5] | 2 [1 - 5] | 0.414 |
| SOFA, [IQR] | 2 [1 - 4] | 2 [1 - 4] | 3 [1 - 4] | 0.001 |
| Confirmed Sepsis, % (n) | 76.1 (424/557) | 67.1 (757/1128) | 58.2 (411/706) | < 0.001 |
| Developed Shock, % (n) | 23.7 (132/557) | 22.3 (252/1128) | 19.0 (134/706) | 0.097 |

**Supplemental Table 12:** Baseline Characteristics and Outcomes by Antibiotic Timing from Triage Among Patients Treated with Suspected Sepsis Infection Between Jan 2016 and Dec 2023 at the Development Site in Risk Group 2.

|  | **Antibiotic Timing (From Triage)** | | |  |
| --- | --- | --- | --- | --- |
| **Variables** | **<1 hr** | **1–3 hrs** | **>3 hrs** | **P-Value** |
| Short Term Mortality, % (n) | 20.0 (2/10) | 20.5 (9/44) | 17.5 (10/57) | 0.93 |
| Age, [IQR] | 62 [55 - 77] | 61 [55 - 67] | 56 [48 - 75] | 0.563 |
| Female, % (n) | 30.0 (3/10) | 38.6 (17/44) | 36.8 (21/57) | 0.877 |
| Black Race, % (n) | 0.0 (0/10) | 18.2 (8/44) | 19.3 (11/57) | 0.318 |
| White Race, % (n) | 70.0 (7/10) | 54.5 (24/44) | 49.1 (28/57) | 0.462 |
| Asian Race, % (n) | 10.0 (1/10) | 4.5 (2/44) | 12.3 (7/57) | 0.401 |
| Charlson Comorbidity Index, [IQR] | 0 [0 - 2] | 1 [0 - 4] | 1 [0 - 3] | 0.204 |
| SOFA, [IQR] | 2 [2 - 4] | 3 [2 - 4] | 3 [2 - 6] | 0.38 |
| Confirmed Sepsis, % (n) | 60.0 (6/10) | 68.2 (30/44) | 66.7 (38/57) | 0.885 |
| Developed Shock, % (n) | 50.0 (5/10) | 38.6 (17/44) | 40.4 (23/57) | 0.803 |

**Supplemental Table 13:** Baseline Characteristics and Outcomes by Antibiotic Timing from Triage Among Patients Treated with Suspected Sepsis Infection Between Jan 2016 and Dec 2023 at the Development Site in Risk Group 3.

|  | **Antibiotic Timing (From Triage)** | | |  |
| --- | --- | --- | --- | --- |
| **Variables** | **<1 hr** | **1–3 hrs** | **>3 hrs** | **P-Value** |
| Short Term Mortality, % (n) | 4.2 (77/1820) | 5.6 (288/5164) | 5.9 (349/5920) | 0.025 |
| Age, [IQR] | 62 [49 - 72] | 62 [49 - 73] | 61 [48 - 72] | 0.154 |
| Female, % (n) | 33.9 (617/1820) | 38.7 (2001/5164) | 42.5 (2516/5920) | < 0.001 |
| Black Race, % (n) | 10.5 (192/1820) | 10.9 (562/5164) | 11.5 (681/5920) | 0.412 |
| White Race, % (n) | 50.1 (912/1820) | 52.1 (2691/5164) | 50.8 (3007/5920) | 0.227 |
| Asian Race, % (n) | 9.2 (167/1820) | 7.8 (401/5164) | 8.0 (472/5920) | 0.155 |
| Charlson Comorbidity Index, [IQR] | 2 [1 - 5] | 2 [1 - 5] | 2 [1 - 5] | 0.004 |
| SOFA, [IQR] | 1 [0 - 2] | 1 [0 - 2] | 1 [0 - 2] | < 0.001 |
| Confirmed Sepsis, % (n) | 52.4 (953/1820) | 44.6 (2303/5164) | 34.8 (2058/5920) | < 0.001 |
| Developed Shock, % (n) | 9.9 (181/1820) | 8.4 (432/5164) | 4.6 (275/5920) | < 0.001 |

**Supplemental Table 14:** Baseline Characteristics and Outcomes by Antibiotic Timing from Triage Among Patients Treated with Suspected Sepsis Infection Between Jan 2016 and Dec 2023 at the Development Site in Risk Group 4.

|  | **Antibiotic Timing (From Triage)** | | |  |
| --- | --- | --- | --- | --- |
| **Variables** | **<1 hr** | **1–3 hrs** | **>3 hrs** | **P-Value** |
| Short Term Mortality, % (n) | 1.0 (4/396) | 2.4 (51/2091) | 2.4 (144/6020) | 0.199 |
| Age, [IQR] | 56 [40 - 67] | 58 [43 - 69] | 59 [43 - 70] | 0.012 |
| Female, % (n) | 46.5 (184/396) | 43.7 (913/2091) | 49.0 (2952/6020) | < 0.001 |
| Black Race, % (n) | 13.4 (53/396) | 13.4 (280/2091) | 12.8 (769/6020) | 0.744 |
| White Race, % (n) | 51.3 (203/396) | 51.0 (1066/2091) | 51.8 (3116/6020) | 0.822 |
| Asian Race, % (n) | 5.8 (23/396) | 7.6 (158/2091) | 6.7 (402/6020) | 0.274 |
| Charlson Comorbidity Index, [IQR] | 1 [0 - 3] | 2 [0 - 4] | 2 [0 - 4] | 0.006 |
| SOFA, [IQR] | 0 [0 - 1] | 0 [0 - 1] | 0 [0 - 1] | 0.042 |
| Confirmed Sepsis, % (n) | 25.5 (101/396) | 22.9 (478/2091) | 17.9 (1075/6020) | < 0.001 |
| Developed Shock, % (n) | 2.3 (9/396) | 3.1 (64/2091) | 1.8 (109/6020) | 0.003 |

**Supplemental Table 15:** Baseline Characteristics and Outcomes by Antibiotic Timing from Triage Among Patients Treated with Suspected Sepsis Infection Between Jan 2023 and Oct 2024 at the Validation Site in Risk Group 1.

|  | **Antibiotic Timing (From Triage)** | | |  |
| --- | --- | --- | --- | --- |
| **Variables** | **<1 hr** | **1–3 hrs** | **>3 hrs** | **P-Value** |
| Short Term Mortality, % (n) | 26.4 (46/174) | 30.8 (92/299) | 27.4 (54/197) | 0.543 |
| Age, [IQR] | 67 [59 - 80] | 68 [55 - 78] | 65 [54 - 77] | 0.393 |
| Female, % (n) | 40.8 (71/174) | 44.8 (134/299) | 44.7 (88/197) | 0.664 |
| Black Race, % (n) | 0.6 (1/174) | 1.3 (4/299) | 2.0 (4/197) | 0.478 |
| White Race, % (n) | 39.7 (69/174) | 45.2 (135/299) | 45.2 (89/197) | 0.452 |
| Asian Race, % (n) | 28.7 (50/174) | 23.4 (70/299) | 19.8 (39/197) | 0.128 |
| Charlson Comorbidity Index, [IQR] | 2 [0 - 4] | 1 [0 - 3] | 1 [0 - 3] | 0.581 |
| SOFA, [IQR] | 3 [1 - 5] | 3 [1 - 5] | 3 [1 - 5] | 0.506 |
| Confirmed Sepsis, % (n) | 85.6 (149/174) | 75.6 (226/299) | 66.5 (131/197) | < 0.001 |
| Developed Shock, % (n) | 47.7 (83/174) | 41.5 (124/299) | 28.9 (57/197) | < 0.001 |

**Supplemental Table 16:** Baseline Characteristics and Outcomes by Antibiotic Timing from Triage Among Patients Treated with Suspected Sepsis Infection Between Jan 2023 and Oct 2024 at the Validation Site in Risk Group 2.

|  | **Antibiotic Timing (From Triage)** | | |  |
| --- | --- | --- | --- | --- |
| **Variables** | **<1 hr** | **1–3 hrs** | **>3 hrs** | **P-Value** |
| Short Term Mortality, % (n) | 20.0 (1/5) | 22.2 (4/18) | 12.5 (3/24) | 0.696 |
| Age, [IQR] | 55 [45 - 69] | 67 [49 - 79] | 69 [61 - 78] | 0.686 |
| Female, % (n) | 0.0 (0/5) | 44.4 (8/18) | 37.5 (9/24) | 0.184 |
| Black Race, % (n) | 0.0 (0/5) | 0.0 (0/18) | 4.2 (1/24) | 0.613 |
| White Race, % (n) | 20.0 (1/5) | 38.9 (7/18) | 54.2 (13/24) | 0.309 |
| Asian Race, % (n) | 40.0 (2/5) | 44.4 (8/18) | 16.7 (4/24) | 0.13 |
| Charlson Comorbidity Index, [IQR] | 0 [0 - 0] | 0 [0 - 2] | 1 [0 - 3] | 0.483 |
| SOFA, [IQR] | 0 [0 - 5] | 2 [1 - 4] | 3 [2 - 5] | 0.234 |
| Confirmed Sepsis, % (n) | 40.0 (2/5) | 66.7 (12/18) | 33.3 (8/24) | 0.096 |
| Developed Shock, % (n) | 40.0 (2/5) | 27.8 (5/18) | 12.5 (3/24) | 0.272 |

**Supplemental Table 17:** Baseline Characteristics and Outcomes by Antibiotic Timing from Triage Among Patients Treated with Suspected Sepsis Infection Between Jan 2023 and Oct 2024 at the Validation Site in Risk Group 3.

|  | **Antibiotic Timing (From Triage)** | | |  |
| --- | --- | --- | --- | --- |
| **Variables** | **<1 hr** | **1–3 hrs** | **>3 hrs** | **P-Value** |
| Short Term Mortality, % (n) | 6.7 (12/179) | 8.7 (40/461) | 9.2 (82/893) | 0.562 |
| Age, [IQR] | 53 [33 - 68] | 62 [44 - 76] | 60 [44 - 72] | < 0.001 |
| Female, % (n) | 38.5 (69/179) | 44.9 (207/461) | 44.5 (397/893) | 0.304 |
| Black Race, % (n) | 2.2 (4/179) | 1.5 (7/461) | 3.1 (28/893) | 0.194 |
| White Race, % (n) | 39.7 (71/179) | 44.9 (207/461) | 45.0 (402/893) | 0.405 |
| Asian Race, % (n) | 20.1 (36/179) | 24.1 (111/461) | 18.8 (168/893) | 0.075 |
| Charlson Comorbidity Index, [IQR] | 0 [0 - 2] | 1 [0 - 3] | 1 [0 - 4] | < 0.001 |
| SOFA, [IQR] | 1 [0 - 2] | 1 [0 - 2] | 1 [0 - 2] | 0.69 |
| Confirmed Sepsis, % (n) | 34.1 (61/179) | 46.0 (212/461) | 33.4 (298/893) | < 0.001 |
| Developed Shock, % (n) | 5.6 (10/179) | 7.8 (36/461) | 4.4 (39/893) | 0.032 |

**Supplemental Table 18:** Baseline Characteristics and Outcomes by Antibiotic Timing from Triage Among Patients Treated with Suspected Sepsis Infection Between Jan 2023 and Oct 2024 at the Validation Site in Risk Group 4.

|  | **Antibiotic Timing (From Triage)** | | |  |
| --- | --- | --- | --- | --- |
| **Variables** | **<1 hr** | **1–3 hrs** | **>3 hrs** | **P-Value** |
| Short Term Mortality, % (n) | 4.1 (5/122) | 5.0 (14/279) | 2.7 (35/1310) | 0.104 |
| Age, [IQR] | 52 [32 - 67] | 59 [37 - 76] | 56 [37 - 71] | 0.032 |
| Female, % (n) | 32.8 (40/122) | 45.9 (128/279) | 49.5 (648/1310) | 0.002 |
| Black Race, % (n) | 4.9 (6/122) | 3.2 (9/279) | 3.6 (47/1310) | 0.699 |
| White Race, % (n) | 44.3 (54/122) | 46.2 (129/279) | 46.1 (604/1310) | 0.923 |
| Asian Race, % (n) | 13.9 (17/122) | 21.5 (60/279) | 16.4 (215/1310) | 0.077 |
| Charlson Comorbidity Index, [IQR] | 0 [0 - 1] | 1 [0 - 2] | 1 [0 - 3] | < 0.001 |
| SOFA, [IQR] | 0 [0 - 1] | 0 [0 - 1] | 0 [0 - 1] | 0.958 |
| Confirmed Sepsis, % (n) | 13.1 (16/122) | 22.2 (62/279) | 13.1 (172/1310) | < 0.001 |
| Developed Shock, % (n) | 3.3 (4/122) | 3.6 (10/279) | 0.8 (10/1310) | < 0.001 |

**Supplemental Table 19:** Baseline Characteristics and Outcomes by Antibiotic Timing from Triage Among Patients Treated with Suspected Sepsis Infection Between Jan 2016 and Dec 2023 at the Development Site in Risk Group 1. Patients who develop shock within 3 hours of triage are excluded from this analysis.

|  | **Antibiotic Timing (From Triage)** | | |  |
| --- | --- | --- | --- | --- |
| **Variables** | **<1 hr** | **1–3 hrs** | **>3 hrs** | **P-Value** |
| Short Term Mortality, % (n) | 14.2 (79/557) | 19.1 (215/1128) | 18.7 (132/706) | 0.037 |
| Age, [IQR] | 67 [55 - 76] | 64 [54 - 75] | 63 [52 - 75] | 0.01 |
| Female, % (n) | 37.0 (206/557) | 39.6 (447/1128) | 43.6 (308/706) | 0.05 |
| Black Race, % (n) | 8.3 (46/557) | 9.4 (106/1128) | 10.2 (72/706) | 0.501 |
| White Race, % (n) | 53.3 (297/557) | 53.2 (600/1128) | 49.0 (346/706) | 0.168 |
| Asian Race, % (n) | 9.9 (55/557) | 8.2 (92/1128) | 7.9 (56/706) | 0.403 |
| Charlson Comorbidity Index, [IQR] | 2 [1 - 5] | 2 [1 - 5] | 2 [1 - 5] | 0.414 |
| SOFA, [IQR] | 2 [1 - 4] | 2 [1 - 4] | 3 [1 - 4] | 0.001 |
| Confirmed Sepsis, % (n) | 76.1 (424/557) | 67.1 (757/1128) | 58.2 (411/706) | < 0.001 |
| Developed Shock, % (n) | 23.7 (132/557) | 22.3 (252/1128) | 19.0 (134/706) | 0.097 |

**Supplemental Table 20:** Baseline Characteristics and Outcomes by Antibiotic Timing from Triage Among Patients Treated with Suspected Sepsis Infection Between Jan 2016 and Dec 2023 at the Development Site in Risk Group 2. Patients who develop shock within 3 hours of triage are excluded from this analysis.

|  | **Antibiotic Timing (From Triage)** | | |  |
| --- | --- | --- | --- | --- |
| **Variables** | **<1 hr** | **1–3 hrs** | **>3 hrs** | **P-Value** |
| Short Term Mortality, % (n) | 16.7 (1/6) | 16.7 (5/30) | 13.3 (6/45) | 0.916 |
| Age, [IQR] | 62 [56 - 73] | 61 [55 - 66] | 60 [48 - 77] | 0.885 |
| Female, % (n) | 16.7 (1/6) | 43.3 (13/30) | 42.2 (19/45) | 0.457 |
| Black Race, % (n) | 0.0 (0/6) | 16.7 (5/30) | 20.0 (9/45) | 0.474 |
| White Race, % (n) | 83.3 (5/6) | 56.7 (17/30) | 48.9 (22/45) | 0.268 |
| Asian Race, % (n) | 0.0 (0/6) | 6.7 (2/30) | 11.1 (5/45) | 0.588 |
| Charlson Comorbidity Index, [IQR] | 0 [0 - 2] | 1 [0 - 3] | 2 [0 - 3] | 0.282 |
| SOFA, [IQR] | 2 [1 - 4] | 3 [2 - 4] | 3 [1 - 4] | 0.697 |
| Confirmed Sepsis, % (n) | 33.3 (2/6) | 53.3 (16/30) | 57.8 (26/45) | 0.524 |
| Developed Shock, % (n) | 16.7 (1/6) | 10.0 (3/30) | 24.4 (11/45) | 0.286 |

**Supplemental Table 21:** Baseline Characteristics and Outcomes by Antibiotic Timing from Triage Among Patients Treated with Suspected Sepsis Infection Between Jan 2016 and Dec 2023 at the Development Site in Risk Group 3. Patients who develop shock within 3 hours of triage are excluded from this analysis.

|  | **Antibiotic Timing (From Triage)** | | |  |
| --- | --- | --- | --- | --- |
| **Variables** | **<1 hr** | **1–3 hrs** | **>3 hrs** | **P-Value** |
| Short Term Mortality, % (n) | 3.7 (64/1730) | 5.1 (253/5003) | 5.6 (328/5827) | 0.006 |
| Age, [IQR] | 62 [49 - 72] | 62 [49 - 73] | 61 [48 - 72] | 0.185 |
| Female, % (n) | 33.6 (582/1730) | 38.8 (1939/5003) | 42.5 (2475/5827) | < 0.001 |
| Black Race, % (n) | 10.6 (184/1730) | 11.0 (550/5003) | 11.6 (676/5827) | 0.428 |
| White Race, % (n) | 50.3 (870/1730) | 52.0 (2602/5003) | 50.8 (2959/5827) | 0.318 |
| Asian Race, % (n) | 9.1 (157/1730) | 7.7 (383/5003) | 7.9 (461/5827) | 0.167 |
| Charlson Comorbidity Index, [IQR] | 2 [1 - 5] | 2 [1 - 5] | 2 [1 - 5] | 0.005 |
| SOFA, [IQR] | 1 [0 - 2] | 1 [0 - 2] | 1 [0 - 2] | < 0.001 |
| Confirmed Sepsis, % (n) | 49.9 (863/1730) | 42.8 (2142/5003) | 33.7 (1965/5827) | < 0.001 |
| Developed Shock, % (n) | 5.3 (91/1730) | 5.4 (271/5003) | 3.1 (182/5827) | < 0.001 |

**Supplemental Table 22:** Baseline Characteristics and Outcomes by Antibiotic Timing from Triage Among Patients Treated with Suspected Sepsis Infection Between Jan 2016 and Dec 2023 at the Development Site in Risk Group 4. Patients who develop shock within 3 hours of triage are excluded from this analysis.

|  | **Antibiotic Timing (From Triage)** | | |  |
| --- | --- | --- | --- | --- |
| **Variables** | **<1 hr** | **1–3 hrs** | **>3 hrs** | **P-Value** |
| Short Term Mortality, % (n) | 1.0 (4/393) | 2.3 (48/2064) | 2.3 (137/5983) | 0.244 |
| Age, [IQR] | 56 [40 - 67] | 58 [42 - 69] | 59 [43 - 70] | 0.012 |
| Female, % (n) | 46.6 (183/393) | 43.8 (903/2064) | 49.1 (2937/5983) | < 0.001 |
| Black Race, % (n) | 13.5 (53/393) | 13.5 (278/2064) | 12.7 (760/5983) | 0.633 |
| White Race, % (n) | 51.7 (203/393) | 51.2 (1056/2064) | 51.8 (3101/5983) | 0.872 |
| Asian Race, % (n) | 5.6 (22/393) | 7.5 (155/2064) | 6.7 (401/5983) | 0.276 |
| Charlson Comorbidity Index, [IQR] | 1 [0 - 3] | 2 [0 - 4] | 2 [0 - 4] | 0.005 |
| SOFA, [IQR] | 0 [0 - 1] | 0 [0 - 1] | 0 [0 - 1] | 0.028 |
| Confirmed Sepsis, % (n) | 24.9 (98/393) | 21.9 (451/2064) | 17.3 (1038/5983) | < 0.001 |
| Developed Shock, % (n) | 1.5 (6/393) | 1.8 (37/2064) | 1.2 (72/5983) | 0.132 |

**Supplemental Table 23:** Baseline Characteristics and Outcomes by Antibiotic Timing from Triage Among Patients Treated with Suspected Sepsis Infection Between Jan 2023 and Oct 2024 at the Validation Site in Risk Group 1. Patients who develop shock within 3 hours of triage are excluded from this analysis.

|  | **Antibiotic Timing (From Triage)** | | |  |
| --- | --- | --- | --- | --- |
| **Variables** | **<1 hr** | **1–3 hrs** | **>3 hrs** | **P-Value** |
| Short Term Mortality, % (n) | 22.3 (25/112) | 26.4 (55/208) | 22.1 (36/163) | 0.554 |
| Age, [IQR] | 68 [59 - 81] | 70 [55 - 78] | 65 [54 - 78] | 0.359 |
| Female, % (n) | 40.2 (45/112) | 43.3 (90/208) | 47.2 (77/163) | 0.496 |
| Black Race, % (n) | 0.0 (0/112) | 1.0 (2/208) | 2.5 (4/163) | 0.174 |
| White Race, % (n) | 39.3 (44/112) | 47.6 (99/208) | 42.3 (69/163) | 0.319 |
| Asian Race, % (n) | 32.1 (36/112) | 22.1 (46/208) | 20.9 (34/163) | 0.069 |
| Charlson Comorbidity Index, [IQR] | 1 [0 - 3] | 1 [0 - 3] | 1 [1 - 4] | 0.192 |
| SOFA, [IQR] | 2 [1 - 3] | 2 [1 - 4] | 2 [1 - 4] | 0.163 |
| Confirmed Sepsis, % (n) | 77.7 (87/112) | 64.9 (135/208) | 59.5 (97/163) | 0.007 |
| Developed Shock, % (n) | 18.8 (21/112) | 15.9 (33/208) | 14.1 (23/163) | 0.586 |

**Supplemental Table 24:** Baseline Characteristics and Outcomes by Antibiotic Timing from Triage Among Patients Treated with Suspected Sepsis Infection Between Jan 2023 and Oct 2024 at the Validation Site in Risk Group 2. Patients who develop shock within 3 hours of triage are excluded from this analysis.

|  | **Antibiotic Timing (From Triage)** | | |  |
| --- | --- | --- | --- | --- |
| **Variables** | **<1 hr** | **1–3 hrs** | **>3 hrs** | **P-Value** |
| Short Term Mortality, % (n) | 0.0 (0/4) | 7.1 (1/14) | 13.0 (3/23) | 0.663 |
| Age, [IQR] | 62 [50 - 75] | 73 [50 - 82] | 68 [59 - 77] | 0.732 |
| Female, % (n) | 0.0 (0/4) | 35.7 (5/14) | 34.8 (8/23) | 0.357 |
| Black Race, % (n) | 0.0 (0/4) | 0.0 (0/14) | 4.3 (1/23) | 0.67 |
| White Race, % (n) | 25.0 (1/4) | 35.7 (5/14) | 52.2 (12/23) | 0.449 |
| Asian Race, % (n) | 50.0 (2/4) | 57.1 (8/14) | 17.4 (4/23) | 0.037 |
| Charlson Comorbidity Index, [IQR] | 0 [0 - 1] | 0 [0 - 2] | 1 [0 - 3] | 0.747 |
| SOFA, [IQR] | 0 [0 - 1] | 2 [1 - 3] | 3 [2 - 4] | 0.072 |
| Confirmed Sepsis, % (n) | 25.0 (1/4) | 57.1 (8/14) | 30.4 (7/23) | 0.226 |
| Developed Shock, % (n) | 25.0 (1/4) | 7.1 (1/14) | 8.7 (2/23) | 0.551 |

**Supplemental Table 25:** Baseline Characteristics and Outcomes by Antibiotic Timing from Triage Among Patients Treated with Suspected Sepsis Infection Between Jan 2023 and Oct 2024 at the Validation Site in Risk Group 3. Patients who develop shock within 3 hours of triage are excluded from this analysis.

|  | **Antibiotic Timing (From Triage)** | | |  |
| --- | --- | --- | --- | --- |
| **Variables** | **<1 hr** | **1–3 hrs** | **>3 hrs** | **P-Value** |
| Short Term Mortality, % (n) | 6.4 (11/172) | 7.6 (34/450) | 9.1 (80/882) | 0.4 |
| Age, [IQR] | 52 [33 - 68] | 62 [43 - 76] | 60 [44 - 72] | < 0.001 |
| Female, % (n) | 37.2 (64/172) | 44.9 (202/450) | 44.8 (395/882) | 0.167 |
| Black Race, % (n) | 2.3 (4/172) | 1.6 (7/450) | 3.1 (27/882) | 0.25 |
| White Race, % (n) | 40.7 (70/172) | 45.3 (204/450) | 45.2 (399/882) | 0.525 |
| Asian Race, % (n) | 19.2 (33/172) | 24.2 (109/450) | 18.7 (165/882) | 0.056 |
| Charlson Comorbidity Index, [IQR] | 0 [0 - 2] | 1 [0 - 3] | 1 [0 - 4] | < 0.001 |
| SOFA, [IQR] | 1 [0 - 2] | 1 [0 - 2] | 1 [0 - 2] | 0.411 |
| Confirmed Sepsis, % (n) | 31.4 (54/172) | 44.7 (201/450) | 32.5 (287/882) | < 0.001 |
| Developed Shock, % (n) | 1.7 (3/172) | 5.6 (25/450) | 3.2 (28/882) | 0.033 |

**Supplemental Table 26:** Baseline Characteristics and Outcomes by Antibiotic Timing from Triage Among Patients Treated with Suspected Sepsis Infection Between Jan 2023 and Oct 2024 at the Validation Site in Risk Group 4. Patients who develop shock within 3 hours of triage are excluded from this analysis.

|  | **Antibiotic Timing (From Triage)** | | |  |
| --- | --- | --- | --- | --- |
| **Variables** | **<1 hr** | **1–3 hrs** | **>3 hrs** | **P-Value** |
| Short Term Mortality, % (n) | 4.1 (5/121) | 4.3 (12/277) | 2.6 (34/1305) | 0.232 |
| Age, [IQR] | 52 [32 - 67] | 59 [36 - 76] | 56 [37 - 71] | 0.025 |
| Female, % (n) | 32.2 (39/121) | 45.5 (126/277) | 49.5 (646/1305) | < 0.001 |
| Black Race, % (n) | 5.0 (6/121) | 3.2 (9/277) | 3.6 (47/1305) | 0.696 |
| White Race, % (n) | 43.8 (53/121) | 46.2 (128/277) | 46.1 (601/1305) | 0.888 |
| Asian Race, % (n) | 14.0 (17/121) | 21.3 (59/277) | 16.4 (214/1305) | 0.095 |
| Charlson Comorbidity Index, [IQR] | 0 [0 - 1] | 1 [0 - 2] | 1 [0 - 3] | < 0.001 |
| SOFA, [IQR] | 0 [0 - 1] | 0 [0 - 1] | 0 [0 - 1] | 0.925 |
| Confirmed Sepsis, % (n) | 12.4 (15/121) | 21.7 (60/277) | 12.8 (167/1305) | < 0.001 |
| Developed Shock, % (n) | 2.5 (3/121) | 2.9 (8/277) | 0.4 (5/1305) | < 0.001 |

Multivariate Logistic Regression Odds Ratios. P-Value, and E-Values:

**Supplemental Table 27:** Adjusted Odds Ratios for Mortality Associated with Antibiotic Administration ≥1 Hour from Triage by Risk Group in Patients Treated for Suspected Sepsis Between Jan 2016 and Dec 2023 at the Development Site.

|  | **Group 1** | | | **Group 2** | | | **Group 3** | | | **Group 4** | | |
| --- | --- | --- | --- | --- | --- | --- | --- | --- | --- | --- | --- | --- |
| **Variables** | **Odds Ratio (95% CI)** | **p-value** | **E-value** | **Odds Ratio (95% CI)** | **p-value** | **E-value** | **Odds Ratio (95% CI)** | **p-value** | **E-value** | **Odds Ratio (95% CI)** | **p-value** | **E-value** |
| Intercept (baseline risk) | 0.04 (0.03–0.07) | < 0.001 | 44 | 0.04 (0.00–0.70) | 0.028 | 47.63 | 0.01 (0.01–0.02) | < 0.001 | 197.5 | 0.00 (0.00–0.00) | < 0.001 | 2723.79 |
| Antibiotics ≥1 hr (ref: <1 hr) | 1.29 (1.07–1.56) | 0.008 | 1.91 | 1.02 (0.18–5.65) | 0.982 | 1.16 | 1.39 (1.09–1.77) | 0.008 | 2.12 | 2.15 (0.79–5.84) | 0.132 | 3.73 |
| Male (ref: Female) | 1.07 (0.90–1.26) | 0.465 | 1.33 | 0.99 (0.33–2.98) | 0.981 | 1.13 | 0.83 (0.71–0.97) | 0.019 | 1.7 | 1.15 (0.87–1.54) | 0.332 | 1.57 |
| Age (per year) | 1.01 (1.01–1.02) | < 0.001 | 1.14 | 1.02 (0.99–1.06) | 0.138 | 1.18 | 1.02 (1.01–1.02) | < 0.001 | 1.14 | 1.04 (1.03–1.05) | < 0.001 | 1.23 |
| Charlson Comorbidity Index (per point) | 0.97 (0.95–1.00) | 0.052 | 1.2 | 0.84 (0.63–1.12) | 0.238 | 1.66 | 1.06 (1.04–1.09) | < 0.001 | 1.33 | 1.07 (1.02–1.11) | 0.003 | 1.33 |
| SOFA score (per point) | 1.24 (1.20–1.28) | < 0.001 | 1.78 | 1.15 (0.96–1.38) | 0.142 | 1.56 | 1.28 (1.23–1.34) | < 0.001 | 1.89 | 1.21 (1.10–1.34) | < 0.001 | 1.72 |

**Supplemental Table 28:** Adjusted Odds Ratios for Mortality Associated with Antibiotic Administration ≥3 Hours from Triage by Risk Group in Patients Treated for Suspected Sepsis Between Jan 2016 and Dec 2023 at the Development Site.

|  | **Group 1** | | | **Group 2** | | | **Group 3** | | | **Group 4** | | |
| --- | --- | --- | --- | --- | --- | --- | --- | --- | --- | --- | --- | --- |
| **Variables** | **Odds Ratio (95% CI)** | **p-value** | **E-value** | **Odds Ratio (95% CI)** | **p-value** | **E-value** | **Odds Ratio (95% CI)** | **p-value** | **E-value** | **Odds Ratio (95% CI)** | **p-value** | **E-value** |
| Intercept (baseline risk) | 0.06 (0.04–0.08) | < 0.001 | 35.38 | 0.05 (0.00–0.58) | 0.016 | 38.54 | 0.01 (0.01–0.02) | < 0.001 | 154.24 | 0.00 (0.00–0.00) | < 0.001 | 1337.09 |
| Antibiotics ≥3 hr (ref: <3 hr) | 1.01 (0.84–1.22) | 0.913 | 1.11 | 0.71 (0.26–1.99) | 0.517 | 2.16 | 1.10 (0.95–1.28) | 0.212 | 1.44 | 1.03 (0.75–1.41) | 0.869 | 1.19 |
| Male (ref: Female) | 1.06 (0.89–1.25) | 0.525 | 1.3 | 0.96 (0.31–2.91) | 0.937 | 1.26 | 0.83 (0.71–0.96) | 0.015 | 1.72 | 1.15 (0.87–1.54) | 0.331 | 1.57 |
| Age (per year) | 1.01 (1.01–1.02) | < 0.001 | 1.14 | 1.02 (0.99–1.06) | 0.16 | 1.18 | 1.02 (1.01–1.02) | < 0.001 | 1.14 | 1.04 (1.03–1.05) | < 0.001 | 1.23 |
| Charlson Comorbidity Index (per point) | 0.97 (0.95–1.00) | 0.045 | 1.2 | 0.84 (0.64–1.12) | 0.236 | 1.65 | 1.06 (1.04–1.09) | < 0.001 | 1.32 | 1.07 (1.02–1.11) | 0.003 | 1.33 |
| SOFA score (per point) | 1.24 (1.20–1.28) | < 0.001 | 1.78 | 1.16 (0.97–1.40) | 0.112 | 1.6 | 1.28 (1.23–1.34) | < 0.001 | 1.89 | 1.21 (1.10–1.34) | < 0.001 | 1.72 |

**Supplemental Table 29:** Adjusted Odds Ratios for Mortality Associated with Antibiotic Administration ≥1 Hour from Triage by Risk Group in Patients Treated for Suspected Sepsis Between Jan 2023 and Oct 2024 at the Validation Site.

|  | **Group 1** | | | **Group 2** | | | **Group 3** | | | **Group 4** | | |
| --- | --- | --- | --- | --- | --- | --- | --- | --- | --- | --- | --- | --- |
| **Variables** | **Odds Ratio (95% CI)** | **p-value** | **E-value** | **Odds Ratio (95% CI)** | **p-value** | **E-value** | **Odds Ratio (95% CI)** | **p-value** | **E-value** | **Odds Ratio (95% CI)** | **p-value** | **E-value** |
| Intercept (baseline risk) | 0.04 (0.02–0.11) | < 0.001 | 45.22 | 0.05 (0.00–6.93) | 0.231 | 41.2 | 0.01 (0.00–0.02) | < 0.001 | 234.28 | 0.00 (0.00–0.01) | < 0.001 | 675.33 |
| Antibiotics ≥1 hr (ref: <1 hr) | 1.21 (0.80–1.81) | 0.364 | 1.71 | 1.23 (0.08–19.62) | 0.881 | 1.77 | 1.14 (0.61–2.13) | 0.684 | 1.54 | 0.53 (0.20–1.41) | 0.201 | 3.19 |
| Male (ref: Female) | 0.77 (0.54–1.10) | 0.155 | 1.92 | 1.97 (0.28–13.99) | 0.496 | 3.36 | 1.07 (0.74–1.54) | 0.736 | 1.33 | 0.67 (0.38–1.18) | 0.167 | 2.34 |
| Age (per year) | 1.02 (1.01–1.03) | < 0.001 | 1.17 | 1.00 (0.95–1.05) | 0.996 | 1.01 | 1.03 (1.02–1.04) | < 0.001 | 1.21 | 1.04 (1.02–1.06) | < 0.001 | 1.24 |
| Charlson Comorbidity Index (per point) | 1.04 (0.98–1.10) | 0.213 | 1.23 | 0.63 (0.31–1.27) | 0.197 | 2.56 | 1.04 (0.98–1.11) | 0.18 | 1.25 | 1.10 (1.01–1.20) | 0.038 | 1.43 |
| SOFA score (per point) | 1.21 (1.13–1.30) | < 0.001 | 1.72 | 1.43 (0.98–2.09) | 0.061 | 2.22 | 1.20 (1.09–1.33) | < 0.001 | 1.7 | 1.49 (1.24–1.79) | < 0.001 | 2.34 |

**Supplemental Table 30:** Adjusted Odds Ratios for Mortality Associated with Antibiotic Administration ≥3 Hours from Triage by Risk Group in Patients Treated for Suspected Sepsis Between Jan 2023 and Oct 2024 at the Validation Site.

|  | **Group 1** | | | **Group 2** | | | **Group 3** | | | **Group 4** | | |
| --- | --- | --- | --- | --- | --- | --- | --- | --- | --- | --- | --- | --- |
| **Variables** | **Odds Ratio (95% CI)** | **p-value** | **E-value** | **Odds Ratio (95% CI)** | **p-value** | **E-value** | **Odds Ratio (95% CI)** | **p-value** | **E-value** | **Odds Ratio (95% CI)** | **p-value** | **E-value** |
| Intercept (baseline risk) | 0.05 (0.02–0.13) | < 0.001 | 37.9 | 0.08 (0.00–3.95) | 0.206 | 23.93 | 0.01 (0.00–0.02) | < 0.001 | 230.47 | 0.00 (0.00–0.01) | < 0.001 | 742.17 |
| Antibiotics ≥3 hr (ref: <3 hr) | 0.97 (0.66–1.43) | 0.896 | 1.19 | 0.44 (0.07–2.62) | 0.366 | 3.99 | 1.14 (0.79–1.66) | 0.486 | 1.54 | 0.54 (0.30–0.98) | 0.044 | 3.09 |
| Male (ref: Female) | 0.77 (0.54–1.09) | 0.142 | 1.94 | 2.18 (0.31–15.48) | 0.436 | 3.78 | 1.07 (0.74–1.55) | 0.722 | 1.34 | 0.69 (0.39–1.21) | 0.195 | 2.27 |
| Age (per year) | 1.02 (1.01–1.03) | < 0.001 | 1.17 | 1.00 (0.95–1.05) | 0.945 | 1.04 | 1.03 (1.02–1.04) | < 0.001 | 1.21 | 1.04 (1.02–1.06) | < 0.001 | 1.24 |
| Charlson Comorbidity Index (per point) | 1.04 (0.98–1.10) | 0.211 | 1.23 | 0.67 (0.34–1.34) | 0.26 | 2.34 | 1.04 (0.98–1.11) | 0.2 | 1.25 | 1.10 (1.01–1.21) | 0.032 | 1.44 |
| SOFA score (per point) | 1.21 (1.13–1.30) | < 0.001 | 1.72 | 1.44 (1.00–2.07) | 0.049 | 2.24 | 1.20 (1.09–1.33) | < 0.001 | 1.7 | 1.47 (1.22–1.77) | < 0.001 | 2.31 |

**Supplemental Table 31:** Adjusted Odds Ratios for Mortality Associated with Antibiotic Administration ≥1 Hour from Triage by Risk Group in Patients Treated for Suspected Sepsis Between Jan 2016 and Dec 2023 at the Development Site. Patients who develop shock within 3 hours of triage are excluded from this analysis.

|  | **Group 1** | | | **Group 2** | | | **Group 3** | | | **Group 4** | | |
| --- | --- | --- | --- | --- | --- | --- | --- | --- | --- | --- | --- | --- |
| **Variables** | **Odds Ratio (95% CI)** | **p-value** | **E-value** | **Odds Ratio (95% CI)** | **p-value** | **E-value** | **Odds Ratio (95% CI)** | **p-value** | **E-value** | **Odds Ratio (95% CI)** | **p-value** | **E-value** |
| Intercept (baseline risk) | 0.03 (0.02–0.05) | < 0.001 | 66.85 | 0.01 (0.00–1.15) | 0.058 | 134.52 | 0.01 (0.01–0.01) | < 0.001 | 248.37 | 0.00 (0.00–0.00) | < 0.001 | 2827.66 |
| Antibiotics ≥1 hr (ref: <1 hr) | 1.42 (1.08–1.87) | 0.011 | 2.2 | 1.10 (0.09–13.92) | 0.939 | 1.44 | 1.45 (1.11–1.89) | 0.006 | 2.25 | 2.02 (0.74–5.49) | 0.168 | 3.46 |
| Male (ref: Female) | 1.27 (1.01–1.59) | 0.04 | 1.85 | 0.59 (0.12–2.87) | 0.516 | 2.77 | 0.83 (0.71–0.98) | 0.028 | 1.69 | 1.18 (0.88–1.58) | 0.276 | 1.64 |
| Age (per year) | 1.01 (1.01–1.02) | < 0.001 | 1.13 | 1.04 (0.99–1.09) | 0.094 | 1.24 | 1.02 (1.01–1.02) | < 0.001 | 1.15 | 1.04 (1.03–1.05) | < 0.001 | 1.24 |
| Charlson Comorbidity Index (per point) | 1.01 (0.98–1.05) | 0.497 | 1.12 | 0.49 (0.24–1.00) | 0.049 | 3.46 | 1.08 (1.05–1.10) | < 0.001 | 1.37 | 1.07 (1.02–1.12) | 0.003 | 1.34 |
| SOFA score (per point) | 1.23 (1.18–1.29) | < 0.001 | 1.77 | 1.37 (0.99–1.90) | 0.059 | 2.09 | 1.25 (1.20–1.31) | < 0.001 | 1.81 | 1.16 (1.04–1.30) | 0.008 | 1.6 |

**Supplemental Table 32:** Adjusted Odds Ratios for Mortality Associated with Antibiotic Administration ≥3 Hours from Triage by Risk Group in Patients Treated for Suspected Sepsis Between Jan 2016 and Dec 2023 at the Development Site. Patients who develop shock within 3 hours of triage are excluded from this analysis.

|  | **Group 1** | | | **Group 2** | | | **Group 3** | | | **Group 4** | | |
| --- | --- | --- | --- | --- | --- | --- | --- | --- | --- | --- | --- | --- |
| **Variables** | **Odds Ratio (95% CI)** | **p-value** | **E-value** | **Odds Ratio (95% CI)** | **p-value** | **E-value** | **Odds Ratio (95% CI)** | **p-value** | **E-value** | **Odds Ratio (95% CI)** | **p-value** | **E-value** |
| Intercept (baseline risk) | 0.04 (0.02–0.07) | < 0.001 | 49.22 | 0.02 (0.00–0.65) | 0.028 | 107.01 | 0.01 (0.01–0.01) | < 0.001 | 192.27 | 0.00 (0.00–0.00) | < 0.001 | 1471.79 |
| Antibiotics ≥3 hr (ref: <3 hr) | 1.02 (0.81–1.29) | 0.873 | 1.16 | 0.84 (0.20–3.58) | 0.819 | 1.65 | 1.16 (0.98–1.36) | 0.076 | 1.58 | 1.02 (0.74–1.42) | 0.894 | 1.17 |
| Male (ref: Female) | 1.25 (1.00–1.57) | 0.05 | 1.82 | 0.57 (0.12–2.70) | 0.477 | 2.92 | 0.83 (0.71–0.98) | 0.024 | 1.7 | 1.18 (0.88–1.58) | 0.276 | 1.64 |
| Age (per year) | 1.01 (1.01–1.02) | < 0.001 | 1.13 | 1.04 (0.99–1.09) | 0.101 | 1.24 | 1.02 (1.01–1.02) | < 0.001 | 1.15 | 1.04 (1.03–1.05) | < 0.001 | 1.24 |
| Charlson Comorbidity Index (per point) | 1.01 (0.98–1.05) | 0.48 | 1.13 | 0.50 (0.25–1.01) | 0.054 | 3.38 | 1.08 (1.05–1.10) | < 0.001 | 1.37 | 1.07 (1.02–1.12) | 0.003 | 1.34 |
| SOFA score (per point) | 1.24 (1.18–1.29) | < 0.001 | 1.78 | 1.38 (1.00–1.91) | 0.052 | 2.11 | 1.25 (1.20–1.31) | < 0.001 | 1.81 | 1.16 (1.04–1.30) | 0.008 | 1.6 |

**Supplemental Table 33:** Adjusted Odds Ratios for Mortality Associated with Antibiotic Administration ≥1 Hour from Triage by Risk Group in Patients Treated for Suspected Sepsis Between Jan 2023 and Oct 2024 at the Validation Site. Patients who develop shock within 3 hours of triage are excluded from this analysis.

|  | **Group 1** | | | **Group 2** | | | **Group 3** | | | **Group 4** | | |
| --- | --- | --- | --- | --- | --- | --- | --- | --- | --- | --- | --- | --- |
| **Variables** | **Odds Ratio (95% CI)** | **p-value** | **E-value** | **Odds Ratio (95% CI)^a^** | **p-value^a^** | **E-value** | **Odds Ratio (95% CI)** | **p-value** | **E-value** | **Odds Ratio (95% CI)** | **p-value** | **E-value** |
| Intercept (baseline risk) | 0.04 (0.01–0.12) | < 0.001 | 54.39 | 0 | n/a | n/a | 0.01 (0.00–0.02) | < 0.001 | 250.18 | 0.00 (0.00–0.01) | < 0.001 | 801.56 |
| Antibiotics ≥1 hr (ref: <1 hr) | 1.02 (0.60–1.73) | 0.939 | 1.17 | > 1000 | n/a | > 1000 | 1.09 (0.57–2.10) | 0.795 | 1.4 | 0.49 (0.18–1.31) | 0.157 | 3.48 |
| Male (ref: Female) | 0.84 (0.54–1.32) | 0.456 | 1.65 | 157.63 | n/a | 314.76 | 1.03 (0.71–1.51) | 0.862 | 1.22 | 0.76 (0.43–1.35) | 0.344 | 1.97 |
| Age (per year) | 1.02 (1.01–1.04) | 0.001 | 1.17 | 1.12 | n/a | 1.49 | 1.03 (1.02–1.04) | < 0.001 | 1.21 | 1.04 (1.03–1.06) | < 0.001 | 1.25 |
| Charlson Comorbidity Index (per point) | 1.03 (0.96–1.11) | 0.45 | 1.2 | 0.26 | n/a | 7.15 | 1.06 (0.99–1.12) | 0.088 | 1.3 | 1.08 (0.99–1.19) | 0.094 | 1.39 |
| SOFA score (per point) | 1.25 (1.14–1.37) | < 0.001 | 1.81 | 3.17 | n/a | 5.79 | 1.22 (1.10–1.36) | < 0.001 | 1.74 | 1.45 (1.19–1.75) | < 0.001 | 2.25 |

^a^Logistic Regression Model failed due small sample size or complete separation, we employed penalized logistic regression with L1 regularization to obtain stable adjusted odds ratios. This method does not yield p-values or a confidence interval for the Odds Ratio.

**Supplemental Table 34:** Adjusted Odds Ratios for Mortality Associated with Antibiotic Administration ≥3 Hours from Triage by Risk Group in Patients Treated for Suspected Sepsis Between Jan 2023 and Oct 2024 at the Validation Site. Patients who develop shock within 3 hours of triage are excluded from this analysis.

|  | **Group 1** | | | **Group 2** | | | **Group 3** | | | **Group 4** | | |
| --- | --- | --- | --- | --- | --- | --- | --- | --- | --- | --- | --- | --- |
| **Variables** | **Odds Ratio (95% CI)** | **p-value** | **E-value** | **Odds Ratio (95% CI)^a^** | **p-value^a^** | **E-value** | **Odds Ratio (95% CI)** | **p-value** | **E-value** | **Odds Ratio (95% CI)** | **p-value** | **E-value** |
| Intercept (baseline risk) | 0.04 (0.01–0.12) | < 0.001 | 49.08 | 0 | n/a | n/a | 0.01 (0.00–0.02) | < 0.001 | 273.37 | 0.00 (0.00–0.01) | < 0.001 | 992.01 |
| Antibiotics ≥3 hr (ref: <3 hr) | 0.80 (0.50–1.27) | 0.342 | 1.82 | 9.42 | n/a | 18.33 | 1.26 (0.85–1.86) | 0.249 | 1.83 | 0.59 (0.32–1.10) | 0.097 | 2.76 |
| Male (ref: Female) | 0.83 (0.53–1.30) | 0.422 | 1.69 | 25.52 | n/a | 50.54 | 1.04 (0.71–1.52) | 0.831 | 1.25 | 0.77 (0.43–1.38) | 0.386 | 1.9 |
| Age (per year) | 1.02 (1.01–1.04) | 0.001 | 1.17 | 1.07 | n/a | 1.34 | 1.03 (1.02–1.04) | < 0.001 | 1.21 | 1.04 (1.02–1.06) | < 0.001 | 1.25 |
| Charlson Comorbidity Index (per point) | 1.03 (0.96–1.11) | 0.417 | 1.21 | 0.36 | n/a | 5 | 1.05 (0.99–1.12) | 0.111 | 1.29 | 1.08 (0.99–1.19) | 0.091 | 1.39 |
| SOFA score (per point) | 1.26 (1.15–1.38) | < 0.001 | 1.82 | 2.84 | n/a | 5.13 | 1.22 (1.09–1.36) | < 0.001 | 1.73 | 1.44 (1.19–1.74) | < 0.001 | 2.23 |

^a^Logistic Regression Model failed due small sample size or complete separation, we employed penalized logistic regression with L1 regularization to obtain stable adjusted odds ratios. This method does not yield p-values or a confidence interval for the Odds Ratio.

Statistical Comparisons of Short-Term Mortality Rates and Time to Antibiotics Distributions Between Risk Groups:

We applied Shapiro-Wilk and Kolmogorov-Smirnov tests to the time to antibiotics distributions for each risk group at each institution and found that they are all non-normal with p < 0.001 (Supplemental Tables 35-38). We then applied Kruskal-Wallis tests to compare the time to antibiotics distributions of the groups within each institution and found that they are not identical with p < 0.001 (Supplemental Table 39).

We note that the Shapiro-Wilk test is a powerful test for normality, especially with small to moderate sample sizes; It is generally considered ideal for sample sizes under 50. However, it becomes overly sensitive with large sample sizes and in this case, the Kolmogorov-Smirnov test is suggested^3^. We present both statistics in Supplemental Tables 35-38 due to the large variation in sample size between our risk groups.

A Chi-Square test for independence was performed to determine whether short-term mortality rates significantly differed among the four risk groups. The results showed a statistically significant difference (p < 0.001), indicating that at least one group's short-term mortality rate differed from the others (Supplemental Table 40). To identify which specific groups had significant differences, we conducted pairwise Chi-Square post-hoc tests with Bonferroni correction to control for multiple comparisons (Supplemental Tables 41-44). These post-hoc tests revealed that nearly all risk groups did. We found that in some cases the short-term mortality rate of the shock likely to develop and sepsis possible group was not significantly different from some of the other risk groups, likely due to the small size of this group.

We then analyzed the distributions of the time to antibiotics administration from ED triage using a Kruskal-Wallis test. The test yielded a significant result (p < 0.001). To further investigate further which groups had significantly different time-to-antibiotic distributions, we conducted Dunn’s post-hoc pairwise comparisons with Bonferroni correction (Supplemental Tables 45-48). The results indicated that most groups had pairwise significantly different distributions. We found that the time to antibiotics distribution for the shock likely to develop and sepsis possible group was sometimes not significantly different from those in some of the other risk groups.

**Supplemental Table 35**: Normality Test of the Time to Antibiotics Distributions at the Development Site

| Group | Shapiro-Wilk p-value | Kolmogorov-Smirnov p-value | Is Normal? |
| --- | --- | --- | --- |
| Shock Likely to Develop and Sepsis Probable | < 0.001 | < 0.001 | FALSE |
| Shock Likely to Develop and Sepsis Possible | < 0.001 | < 0.001 | FALSE |
| Shock Unlikely to Develop and Sepsis Probable | < 0.001 | < 0.001 | FALSE |
| Shock Unlikely to Develop and Sepsis Possible | < 0.001 | < 0.001 | FALSE |

**Supplemental Table 36:** Normality Test of the Time to Antibiotics Distributions at the Validation Site

| Group | Shapiro-Wilk p-value | Kolmogorov-Smirnov p-value | Is Normal? |
| --- | --- | --- | --- |
| Shock Likely to Develop and Sepsis Probable | < 0.001 | < 0.001 | FALSE |
| Shock Likely to Develop and Sepsis Possible | < 0.001 | < 0.001 | FALSE |
| Shock Unlikely to Develop and Sepsis Probable | < 0.001 | < 0.001 | FALSE |
| Shock Unlikely to Develop and Sepsis Possible | < 0.001 | < 0.001 | FALSE |

**Supplemental Table 37:** Normality Test of the Time to Antibiotics Distributions at the Development Site (Excluding Patients Who Develop Shock Within 3 Hours of ED Triage)

| Group | Shapiro-Wilk p-value | Kolmogorov-Smirnov p-value | Is Normal? |
| --- | --- | --- | --- |
| Shock Likely to Develop and Sepsis Probable | < 0.001 | < 0.001 | FALSE |
| Shock Likely to Develop and Sepsis Possible | < 0.001 | < 0.001 | FALSE |
| Shock Unlikely to Develop and Sepsis Probable | < 0.001 | < 0.001 | FALSE |
| Shock Unlikely to Develop and Sepsis Possible | < 0.001 | < 0.001 | FALSE |

**Supplemental Table 38:** Normality Test of the Time to Antibiotics Distributions at the Validation Site (Excluding Patients Who Develop Shock Within 3 Hours of ED Triage)

| Group | Shapiro-Wilk p-value | Kolmogorov-Smirnov p-value | Is Normal? |
| --- | --- | --- | --- |
| Shock Likely to Develop and Sepsis Probable | < 0.001 | < 0.001 | FALSE |
| Shock Likely to Develop and Sepsis Possible | < 0.001 | < 0.001 | FALSE |
| Shock Unlikely to Develop and Sepsis Probable | < 0.001 | < 0.001 | FALSE |
| Shock Unlikely to Develop and Sepsis Possible | < 0.001 | < 0.001 | FALSE |

**Supplemental Table 39:** Kruskal-Wallace Tests on Time to Antibiotics Distributions of the 4 Risk Groups

| Site | Statistic | P-Value | Conclusion |
| --- | --- | --- | --- |
| Development | 2867 | < 0.001 | At least one group is significantly different |
| Validation | 540 | < 0.001 | At least one group is significantly different |
| Development*^*^* | 2203 | < 0.001 | At least one group is significantly different |
| Validation*^*^* | 400 | < 0.001 | At least one group is significantly different |

* Excluding Patients Who Develop Shock Within 3 Hours of ED Triage

**Supplemental Table 40:** Chi-Squared Tests on the Short-Term Mortality Rates of the 4 risk groups

| Site | Statistic | P-Value | Conclusion |
| --- | --- | --- | --- |
| Development | 2250 | < 0.001 | At least one group is significantly different |
| Validation | 407 | < 0.001 | At least one group is significantly different |
| Development*^*^* | 1226 | < 0.001 | At least one group is significantly different |
| Validation*^*^* | 268 | < 0.001 | At least one group is significantly different |

* Excluding Patients Who Develop Shock Within 3 Hours of ED Triage

**Supplemental Table 41:** Pairwise Chi-Square post-hoc tests with Bonferroni correction on the Short-Term Mortality Rates at the Development Site

| Group A | Group B | P-Value (Unadjusted) | P-Value (Bonferroni Corrected) | Conclusion |
| --- | --- | --- | --- | --- |
| Shock Likely to Develop and Sepsis Probable | Shock Likely to Develop and Sepsis Possible | 0.191 | 1 | Not Significantly Different |
| Shock Likely to Develop and Sepsis Probable | Shock Unlikely to Develop and Sepsis Probable | < 0.001 | < 0.001 | Significantly Different |
| Shock Likely to Develop and Sepsis Probable | Shock Unlikely to Develop and Sepsis Possible | < 0.001 | < 0.001 | Significantly Different |
| Shock Likely to Develop and Sepsis Possible | Shock Unlikely to Develop and Sepsis Probable | < 0.001 | < 0.001 | Significantly Different |
| Shock Likely to Develop and Sepsis Possible | Shock Unlikely to Develop and Sepsis Possible | < 0.001 | < 0.001 | Significantly Different |
| Shock Unlikely to Develop and Sepsis Probable | Shock Unlikely to Develop and Sepsis Possible | < 0.001 | < 0.001 | Significantly Different |

**Supplemental Table 42:** Pairwise Chi-Square Post-Hoc tests with Bonferroni Correction on the Short-Term Mortality Rates at the Validation Site

| Group A | Group B | P-Value (Unadjusted) | P-Value (Bonferroni Corrected) | Conclusion |
| --- | --- | --- | --- | --- |
| Shock Likely to Develop and Sepsis Probable | Shock Likely to Develop and Sepsis Possible | 0.067 | 0.404 | Not Significantly Different |
| Shock Likely to Develop and Sepsis Probable | Shock Unlikely to Develop and Sepsis Probable | < 0.001 | < 0.001 | Significantly Different |
| Shock Likely to Develop and Sepsis Probable | Shock Unlikely to Develop and Sepsis Possible | < 0.001 | < 0.001 | Significantly Different |
| Shock Likely to Develop and Sepsis Possible | Shock Unlikely to Develop and Sepsis Probable | 0.115 | 0.690 | Not Significantly Different |
| Shock Likely to Develop and Sepsis Possible | Shock Unlikely to Develop and Sepsis Possible | < 0.001 | < 0.001 | Significantly Different |
| Shock Unlikely to Develop and Sepsis Probable | Shock Unlikely to Develop and Sepsis Possible | < 0.001 | < 0.001 | Significantly Different |

**Supplemental Table 43:** Pairwise Chi-Square Post-Hoc tests with Bonferroni Correction on the Short-Term Mortality Rates at the Development Site (Excluding Patients Who Develop Shock Within 3 Hours of ED Triage)

| Group A | Group B | P-Value (Unadjusted) | P-Value (Bonferroni Corrected) | Conclusion |
| --- | --- | --- | --- | --- |
| Shock Likely to Develop and Sepsis Probable | Shock Likely to Develop and Sepsis Possible | 0.353 | 1 | Not Significantly Different |
| Shock Likely to Develop and Sepsis Probable | Shock Unlikely to Develop and Sepsis Probable | < 0.001 | < 0.001 | Significantly Different |
| Shock Likely to Develop and Sepsis Probable | Shock Unlikely to Develop and Sepsis Possible | < 0.001 | < 0.001 | Significantly Different |
| Shock Likely to Develop and Sepsis Possible | Shock Unlikely to Develop and Sepsis Probable | < 0.001 | < 0.001 | Significantly Different |
| Shock Likely to Develop and Sepsis Possible | Shock Unlikely to Develop and Sepsis Possible | < 0.001 | < 0.001 | Significantly Different |
| Shock Unlikely to Develop and Sepsis Probable | Shock Unlikely to Develop and Sepsis Possible | < 0.001 | < 0.001 | Significantly Different |

**Supplemental Table 44:** Pairwise Chi-Square Post-Hoc Tests with Bonferroni correction on the Short-Term Mortality Rates at the Validation Site (Excluding Patients Who Develop Shock Within 3 Hours of ED Triage)

| Group A | Group B | P-Value (Unadjusted) | P-Value (Bonferroni Corrected) | Conclusion |
| --- | --- | --- | --- | --- |
| Shock Likely to Develop and Sepsis Probable | Shock Likely to Develop and Sepsis Possible | 0.030 | 0.181 | Not Significantly Different |
| Shock Likely to Develop and Sepsis Probable | Shock Unlikely to Develop and Sepsis Probable | < 0.001 | < 0.001 | Significantly Different |
| Shock Likely to Develop and Sepsis Probable | Shock Unlikely to Develop and Sepsis Possible | < 0.001 | < 0.001 | Significantly Different |
| Shock Likely to Develop and Sepsis Possible | Shock Unlikely to Develop and Sepsis Probable | 1 | 1 | Not Significantly Different |
| Shock Likely to Develop and Sepsis Possible | Shock Unlikely to Develop and Sepsis Possible | 0.052 | 0.315 | Not Significantly Different |
| Shock Unlikely to Develop and Sepsis Probable | Shock Unlikely to Develop and Sepsis Possible | < 0.001 | < 0.001 | Significantly Different |

**Supplemental Table 45:** Dunn’s Post-Hoc Pairwise Comparisons with Bonferroni Correction on the Time to Antibiotics Distributions at the Development Site

| Group A | Group B | P-Value (Bonferroni Corrected) | Conclusion |
| --- | --- | --- | --- |
| Shock Likely to Develop and Sepsis Possible | Shock Likely to Develop and Sepsis Probable | < 0.001 | Significantly Different |
| Shock Likely to Develop and Sepsis Possible | Shock Unlikely to Develop and Sepsis Possible | < 0.001 | Significantly Different |
| Shock Likely to Develop and Sepsis Possible | Shock Unlikely to Develop and Sepsis Probable | 0.959 | Not Significantly Different |
| Shock Likely to Develop and Sepsis Probable | Shock Unlikely to Develop and Sepsis Possible | < 0.001 | Significantly Different |
| Shock Likely to Develop and Sepsis Probable | Shock Unlikely to Develop and Sepsis Probable | < 0.001 | Significantly Different |
| Shock Unlikely to Develop and Sepsis Possible | Shock Unlikely to Develop and Sepsis Probable | < 0.001 | Significantly Different |

**Supplemental Table 46:** Dunn’s Post-Hoc Pairwise Comparisons with Bonferroni Correction on the Time to Antibiotics Distributions at the Validation Site

| Group A | Group B | P-Value (Bonferroni Corrected) | Conclusion |
| --- | --- | --- | --- |
| Shock Likely to Develop and Sepsis Possible | Shock Likely to Develop and Sepsis Probable | 0.006 | Significantly Different |
| Shock Likely to Develop and Sepsis Possible | Shock Unlikely to Develop and Sepsis Possible | 0.001 | Significantly Different |
| Shock Likely to Develop and Sepsis Possible | Shock Unlikely to Develop and Sepsis Probable | 1 | Not Significantly Different |
| Shock Likely to Develop and Sepsis Probable | Shock Unlikely to Develop and Sepsis Possible | < 0.001 | Significantly Different |
| Shock Likely to Develop and Sepsis Probable | Shock Unlikely to Develop and Sepsis Probable | < 0.001 | Significantly Different |
| Shock Unlikely to Develop and Sepsis Possible | Shock Unlikely to Develop and Sepsis Probable | < 0.001 | Significantly Different |

**Supplemental Table 47:** Dunn’s Post-Hoc Pairwise Comparisons with Bonferroni Correction on the Time to Antibiotics Distributions at the Development Site (Excluding Patients Who Develop Shock Within 3 Hours of ED Triage)

| Group A | Group B | P-Value (Bonferroni Corrected) | Conclusion |
| --- | --- | --- | --- |
| Shock Likely to Develop and Sepsis Possible | Shock Likely to Develop and Sepsis Probable | < 0.001 | Significantly Different |
| Shock Likely to Develop and Sepsis Possible | Shock Unlikely to Develop and Sepsis Possible | 0.060 | Not Significantly Different |
| Shock Likely to Develop and Sepsis Possible | Shock Unlikely to Develop and Sepsis Probable | 0.151 | Not Significantly Different |
| Shock Likely to Develop and Sepsis Probable | Shock Unlikely to Develop and Sepsis Possible | < 0.001 | Significantly Different |
| Shock Likely to Develop and Sepsis Probable | Shock Unlikely to Develop and Sepsis Probable | < 0.001 | Significantly Different |
| Shock Unlikely to Develop and Sepsis Possible | Shock Unlikely to Develop and Sepsis Probable | < 0.001 | Significantly Different |

**Supplemental Table 48:** Dunn’s Post-Hoc Pairwise Comparisons with Bonferroni Correction on the Time to Antibiotics Distributions at the Validation Site (Excluding Patients Who Develop Shock Within 3 Hours of ED Triage)

| Group A | Group B | P-Value (Bonferroni Corrected) | Conclusion |
| --- | --- | --- | --- |
| Shock Likely to Develop and Sepsis Possible | Shock Likely to Develop and Sepsis Probable | 0.012 | Significantly Different |
| Shock Likely to Develop and Sepsis Possible | Shock Unlikely to Develop and Sepsis Possible | 0.019 | Significantly Different |
| Shock Likely to Develop and Sepsis Possible | Shock Unlikely to Develop and Sepsis Probable | 1 | Not Significantly Different |
| Shock Likely to Develop and Sepsis Probable | Shock Unlikely to Develop and Sepsis Possible | < 0.001 | Significantly Different |
| Shock Likely to Develop and Sepsis Probable | Shock Unlikely to Develop and Sepsis Probable | < 0.001 | Significantly Different |
| Shock Unlikely to Develop and Sepsis Possible | Shock Unlikely to Develop and Sepsis Probable | < 0.001 | Significantly Different |

**References:**

1. Shashikumar SP, Le JP, Yung N, et al. Development and Validation of a Deep Learning Model for Prediction of Adult Physiological Deterioration. Crit Care Explor. 2024;6(9):e1151. Published 2024 Sep 11. doi:10.1097/CCE.0000000000001151

2. Steinberg DM, Fine J, Chappell R. Sample size for positive and negative predictive value in diagnostic research using case-control designs. Biostatistics. 2009;10(1):94-105. doi:10.1093/biostatistics/kxn018

3. Razali NR, Wah YB. Power comparisons of Shapiro-Wilk, Kolmogorov-Smirnov, Lilliefors and Anderson-Darling tests. J Stat Model Anal. 2011;2(1):21–33.

**Supplementary Figures 1 to 4**


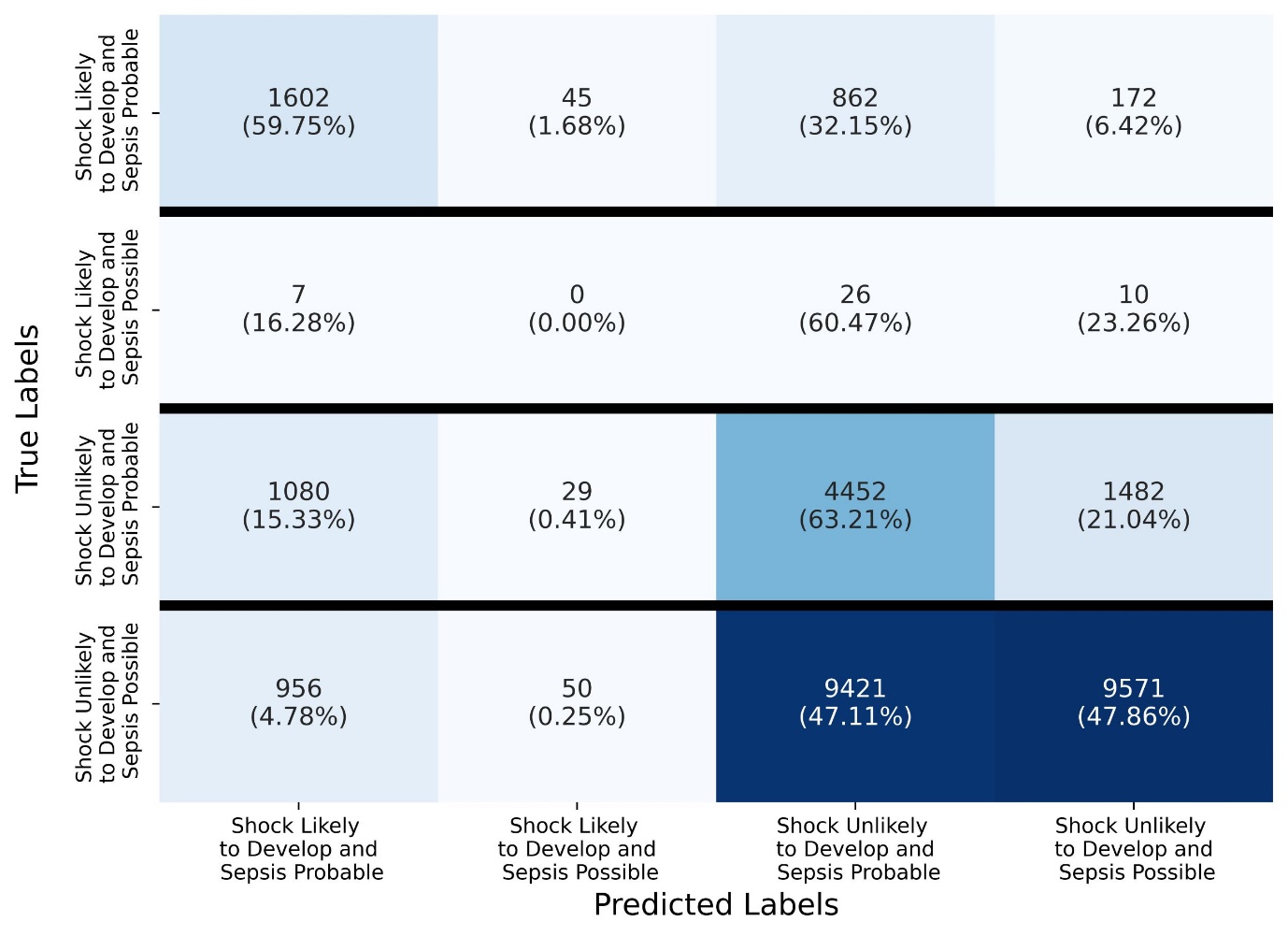


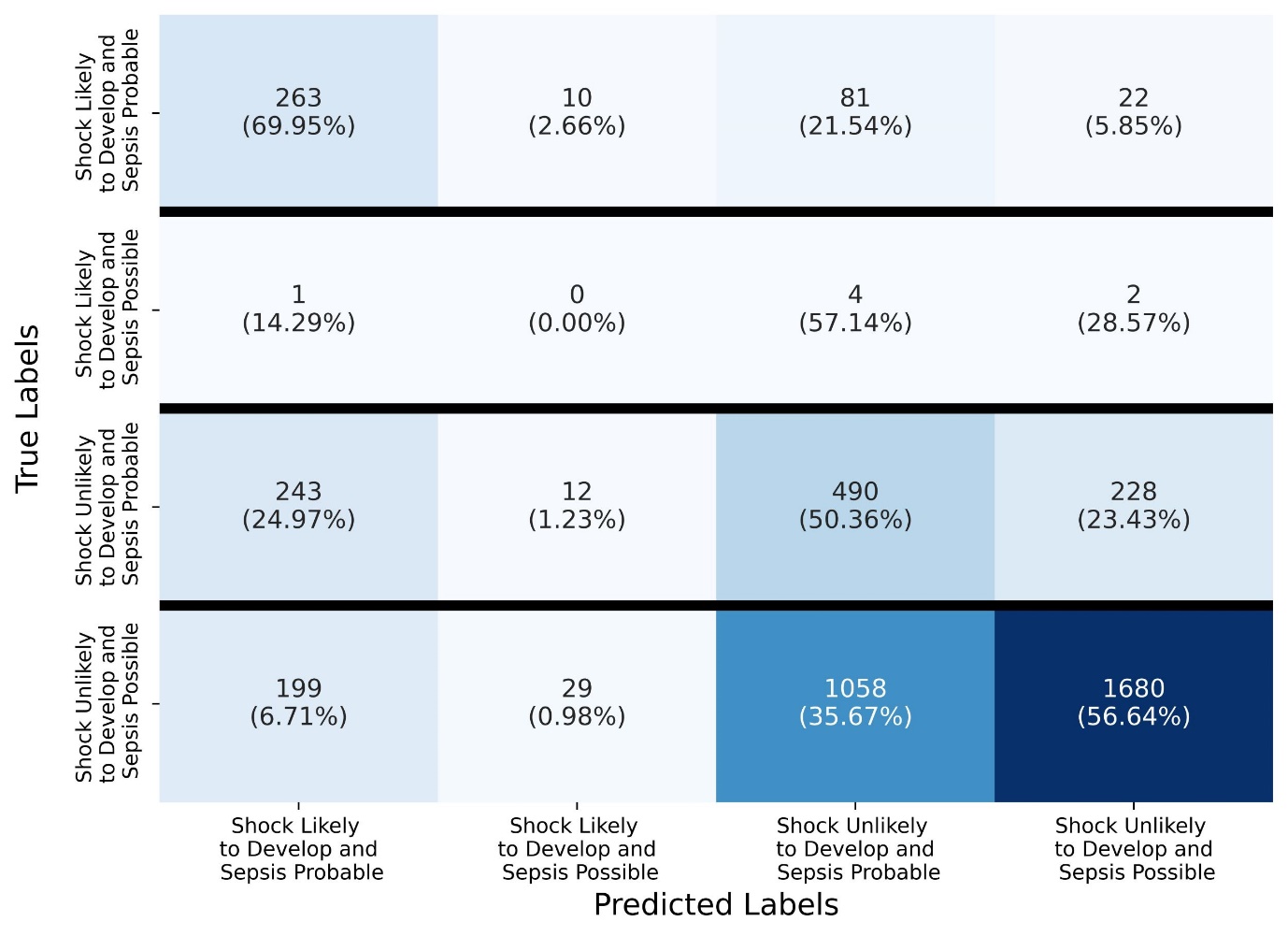


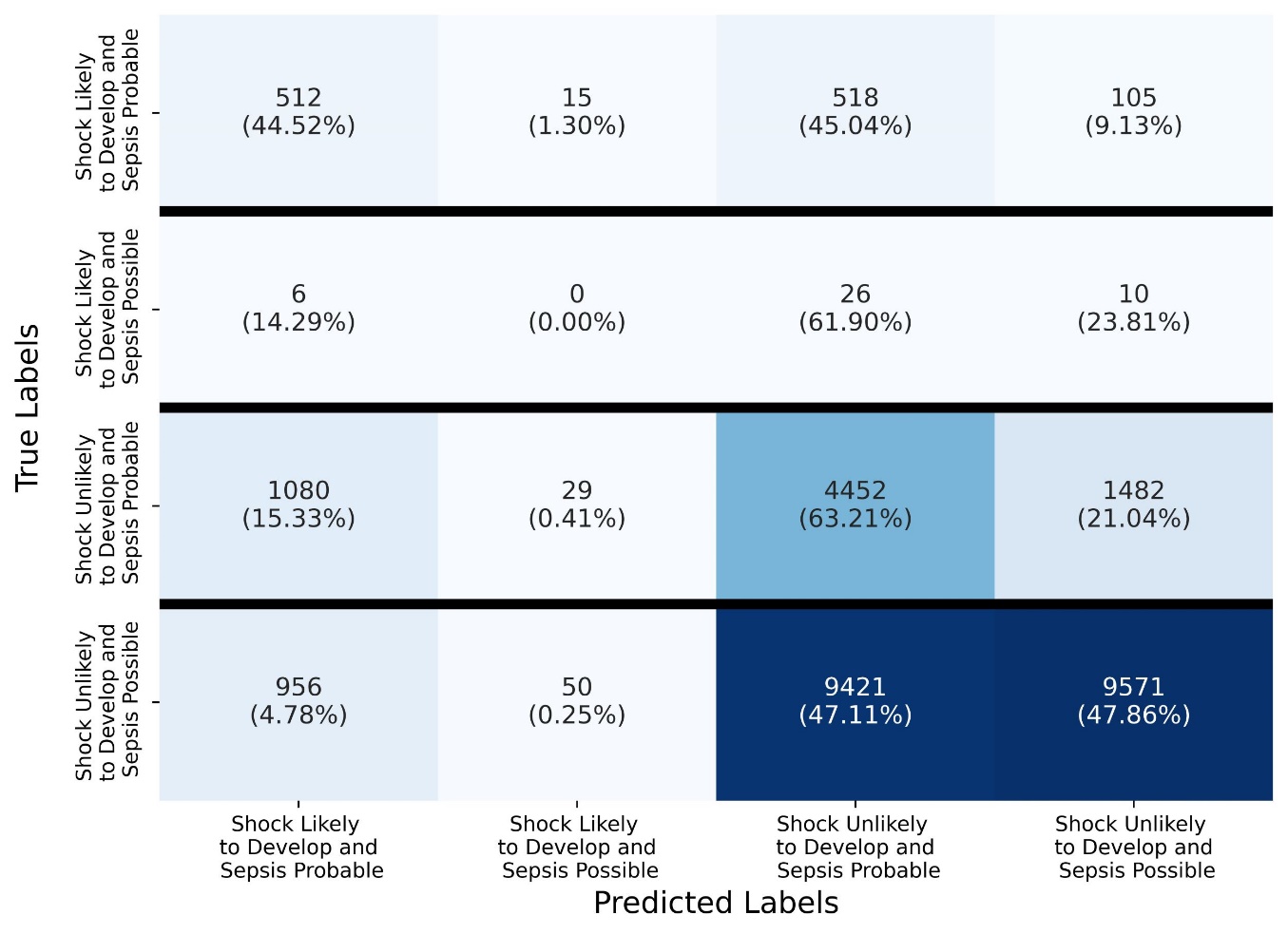


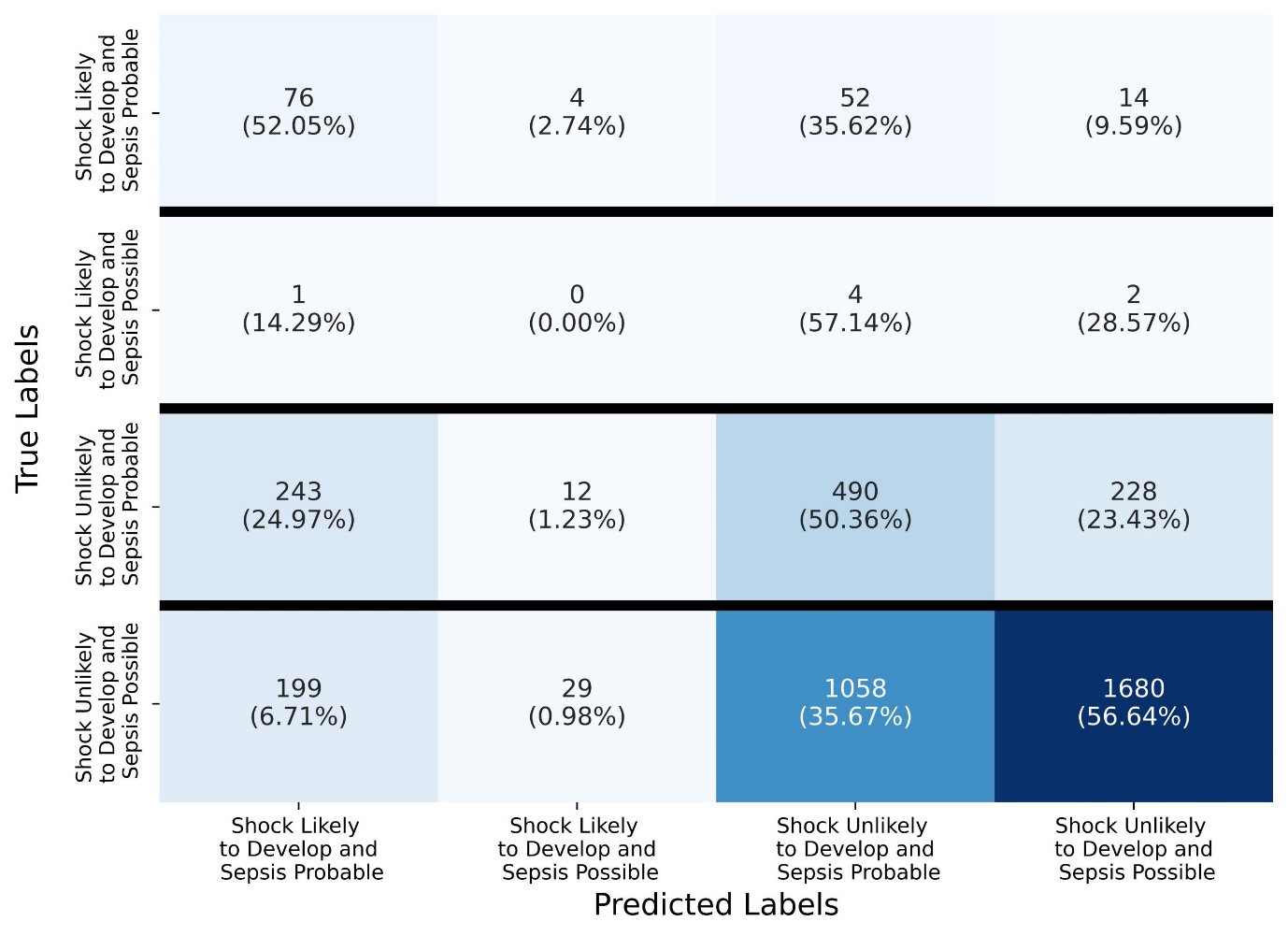

Supplement: Supplementary file 1 — Supplementary Material 1 [file 13054_2025_5493_MOESM1_ESM.docx]
